# Evaluation of the Dimensional Accuracy of Polymeric Helical Gears Manufactured via Fused Filament Fabrication (FFF) †

**DOI:** 10.3390/mi17101199

**Published:** 2026-10-17

**Authors:** Enrique E. Zayas-Figueras, Irene Buj-Corral, Ot Godó-Estany

**Affiliations:** Department of Mechanical Engineering, Barcelona School of Industrial Engineering (ETSEIB), Universtiat Politècnica de Catalunya—Barcelona Tech (UPC), 08028 Barcelona, Spain; enrique.zayas@upc.edu (E.E.Z.-F.); ot.godo@estudiantat.upc.edu (O.G.-E.)

**Keywords:** 3D printing, polymeric helical gears, dimensional accuracy, PLA, PA66, FFF technology, gear measurements

## Abstract

Polymeric gears are commonly employed in applications where low weight and noise, as well as corrosion resistance, are important, such as consumer products, automotive components, and medical devices. The use of additive manufacturing (AM) processes provides greater geometric freedom and lower production costs for short production runs than other manufacturing processes such as injection molding. This study presents an evaluation of the dimensional accuracy of helical gears manufactured using fused filament fabrication (FFF) technology. Three gear models with the same module and number of teeth—but with different helix angles of 15°, 25°, and 35°, respectively—were designed and 3D printed using PLA and PA66. Dimensional verification was performed using two approaches: (i) conventional methods, namely, the tip circle method for measuring tooth thickness, the sector span method, and the over-rollers method for measuring gear diameter; and (ii) a non-contact method, laser scanning, in which dimensional deviations between the theoretical and measured surfaces were determined. The results show that, as a general trend, PA66 gears have higher thickness and lower chordal distance than PLA gears. PA66 parts have a deficit of material at the tooth tip, while PLA gears obtained with *β* = 15° exhibit a slight deficit of material at the bottom part of the tooth space. This study confirms that the combination of conventional and digital measuring methods is appropriate for the dimensional evaluation of 3D-printed polymeric gears.

## 1. Introduction

The advantages offered by polymeric materials, including being lightweight, having an adequate durability, as well as their corrosion resistance, have led to an increase in the use of polymeric materials in several applications, including biomedicine, automobiles, aircraft, ships, etc. For example, polymer gears have unique advantages over metal gears, such as low density, low inertia, high efficiency, quiet operation, the ability to absorb shocks and vibrations as a result of elastic flexibility, the ability to operate with minimal or no lubrication, the possibility of working in environments that are hostile to metal gears, the possibility of using color coding in assembly processes, the wide range of complex geometric shapes and configurations that are possible, the ability to be manufactured with metal inserts and integral designs, etc. [1,2,3].

The most common processes for obtaining polymer gears are (1) plastic injection molding (efficient process for high-volume production of rather small parts, usually made of thermoplastics—polyamides and acetals—and thermoplastic and thermoset composite materials), (2) machining, using almost the same processes as those employed for metal gear machining: turning, milling, drilling, boring, etc. (used to manufacture high-precision gears with larger dimensions) and (3) additive manufacturing (AM), which complements conventional gear manufacturing by offering greater design flexibility and versatility while maintaining quality. In this case, mainly the processes of stereolithography (SLA), selective laser sintering (SLS), fused filament fabrication (FFF), binder jetting (BJ), and material jetting (MJ) are used [1,2,3,4,5,6]. A schematic diagram summarizing the above classification is shown in Figure 1. Figure 2 shows FFF-manufactured polymeric gears having different geometries and colors.

Several authors comment on the many advantages of additive manufacturing (AM) over conventional gear manufacturing, such as (a) relatively high quality and good surface integrity, (b) ability to produce metallic and non-metallic gears, (c) flexibility (freedom of complex geometric shapes), (d) requirement of less tooling and fasteners, (e) efficient use of resources—less material waste—and energy, (f) on-demand production (no stockpiling), and (g) better environmental friendliness, with fewer processing steps and low emissions [5,6]. Other authors argue that AM is particularly important for manufacturing gears with non-standard (non-involute) tooth profiles, like gears with cycloidal tooth profile. In order to manufacture such non-involute gears, it is not possible to use conventional tooth cutting tools; thus, prototypes of such gear wheels are manufactured using AM technologies directly from the created CAD models [7].

AM technologies can be divided into seven different groups, according to the ISO/ASTM 52900 standard [8], including those mentioned in Figure 1 and also Directed Energy Deposition (DED) and Sheet Lamination (SL). Gibson [9] explains that most of them typically follow these steps (Figure 3): (1) 3D modeling of the part with CAD (Computer-Aided Design) software that describes its geometry; (2) Conversion of the 3D model to an STL (Standard Triangle Language) file—which only contains geometric information of the part surface; (3) Manipulation of the STL files using slicer software to ensure the correct size, position, and orientation of the model for manufacturing, as well as to define printing parameters (layer height, support placement, infill rate, etc.); (4) Proper set up of the AM machine prior to the manufacturing process, depending on the technology to be used (calibration of the machine, placement of the printing material, etc.); (5) Building of the part, which is primarily an automated process, allowing the machine to operate largely without supervision; (6) Removal of the part from the machine, once printed, which, depending on the AM technology used, can be a simpler or more complex step; and (7) Post-processing of the part—parts may require additional treatment such as cleaning, surface finishing, support removal, dimension correction, etc., before they are ready for use.

Regarding additively manufactured gears, Vasilescu and Fleser [10] study the production of both spur and helical gears with polylactic acid (PLA), acrylonitrile butadiene styrene (ABS), and polyethylene terephthalate glycol-modified (PETG), recommending PLA because of its higher compressive strength and lower elongation at break. They suggest using a pressure angle of 25° to 35° for 3D-printed gears, instead of the 14.5° and 20° usually employed, as well as choosing a standardized modulus in the modeling software to correct for shrinkage or expansion. They point out that shrinkage is lower in the axial (vertical printing) direction. They propose to assemble two gears coaxially as one piece and offer recommendations for lamination, such as activating support zones to improve adhesion and geometry, adjusting perimeter contours according to the module, and testing different printing configurations (line width, number of layers, etc.). For PLA, they used a printing temperature of 210 °C and a bed temperature of 45 °C. Tunalioglu [11] obtained 3D-printed PLA helical gears. He observed that roughness depended mainly on layer height. During the operation of the part, roughness decreased because of acclimatization and then increased again when the gear’s surface deteriorated.

The usual available methods for the dimensional measurement of gears are contact measurement methods like coordinate measuring machines (CMM) and non-contact measurement methods such as computer tomography (CT) and laser scanning (LS) [12]. In recent years, the availability of laser measurement equipment has enabled the dimensional inspection of additively manufactured parts as an alternative to conventional measurement methods. Laser scanning provides a high number of points in a relatively low measuring time [13]. For example, it has been used to measure material-extrusion [14] and selective-laser-sintered (SLS) parts [13].

As for contact measurement methods for gears, the CMM has been used for the verification of spur and helical gears [15], in which the profile errors, the base thickness error, the errors of flank distortion, and the base pitch error were calculated. This work is based on the ISO 1328 Standard [16], and specific software was developed for this purpose [15]. In a study of 3D-printed ABS and polyamide gears, Masovic et al. found that highest dimensional accuracy corresponded to ABS gears printed in the horizontal position [17]. In order to improve dimensional accuracy of 3D-printed parts, Gao et al. propose two methods: auxiliary heating and polymer modification [18]. Czégé and Ruzicska [19] conducted a geometric characterization of polymeric spur gears fabricated using FFF and SLA processes, employing both traditional analytical methods and a 3D scanner laser technique with an injection-molded polymer gear as a reference. This study shows that FFF gears meet the dimensional accuracy requirements, although their surface roughness is too high for the intended application unless effective post-processing is applied. In contrast, SLA gears exhibit dimensional deviations that exceed acceptable limits, although their surface roughness is adequate. Bodzás and Nemes [20] designed and fabricated a helical gear pair (pinion and gear) in acrylonitrile butadiene styrene (ABS) by means of the FFF technique. They scanned the gears and obtained differences up to 0.15 mm when comparing them to their respective CAD models.

Few works are known on form errors in 3D-printed gears. For example, Dennig et al. [21] reported that poor concentricity could increase pressure on the tool flank of the gears, thus increasing the temperatures of the parts. They also found roundness errors up to 0.674 mm for PA + CF (Polyamide with Carbon Fibre). The roundness error of the inner surface and the tooth profile error of the gears affect the radial clearance of the meshing pair [22]. Consequently, uneven load areas could appear. In hemispherical 3D-printed PLA parts, Luis et al. [23] reported roundness error values up to 0.297 mm and concentricity error values up to 0.272 mm. Gaikwad et al. [24] reported average circularity error of 0.087 mm and average concentricity error of 0.026 mm.

Marciniec et al. [25] measured 3D-printed bevel gears with a blue light scanner and compared them to the CAD file. They configurated the scanner for the measurement of gears, with an increase in data resolution in shortened times and costs. The number of reference points stuck on the part was reduced by using a base rotary table. Measurement of parts with regular axial symmetric shapes (such as gears or toothed pulleys) can lead to measurement errors due to a limited number of reference points. For this reason, the authors suggest to change light intensity, which ensures the covering of the surface of the reflective model, with the use of numerous markers [25].

In order to measure wear of plastic gear material, particularly polyoxymethylene (POM), commonly used in injected molded gears, Yan et al. [26] used online measurement with line-structured light. They measured the surface geometry of rollers in a roller-to-roller friction test, and they compensated data regarding temperature and compensation angle. From these data, they calculated wear. The study enables online measurement of wear volume and provides a detailed analysis of relative wear variations on the component surface for polymeric materials used to manufacture gears. Lu et al. [27] measured orthogonal face gears with 3D optical scanning. A three-stage algorithm is used to compare the measured points and the designed one, including top surfaces, centers of circles, and tooth surfaces. The proposed solution enables more accurate measurements than other methods, such as contact-based techniques. Ciobanu et al. [28] manufactured cylindrical spur gears from PLA using FFF technology, with varying wall thickness. They carried out wear tests and scanned the surface geometry of the gear teeth. The results revealed an inverse relationship between abrasive wear and stiffness. In addition, the tooth tip exhibited greater abrasive wear than the rest of the tooth profile.

Although some authors [19,20,25,26,27,28] have used optical methods to measure additively manufactured parts, little information is available about the comparison between contact and optical dimensional measurements, particularly regarding gears. This study investigates the dimensional characteristics of helical gears fabricated by fused filament fabrication (FFF), which have been scarcely studied in the literature. This study integrates conventional dimensional measurement techniques with laser scanning to establish a methodology for evaluating the dimensional accuracy of 3D-printed helical gears. Specifically, the gears were measured using two approaches: (a) conventional methods, including the tooth thickness measurement, the chordal distance method, and the calibrated rod method; and (b) a non-contact method, namely, laser scanning. The results obtained from both approaches were compared. Three helical gear models with different helix angles were analyzed, namely *β* = 15°, 25°, and 35°, all of them with module *m* = 4 mm and number of teeth *z* = 17. The measured parameters included tooth thickness, sector span, and diametral distance. The present study does not include gear-function tests, profile/lead/pitch deviation measurements, surface roughness, wear testing, torque transmission, nor ISO gear-quality classification.

## 2. Materials and Methods

The methodology used in this study builds upon the approach previously proposed by the authors [1], with two main modifications: (1) CAD modeling of the gears using the SolidWorks^®^ 2025 Toolbox library; and (2) the use of two complementary verification methods: conventional measurement techniques and laser scanning. Thus, the updated methodology comprises 11 distinct steps, which are as follows (Figure 4): (1) Selection of the gear type (helical gear) and its basic parameters (module m, teeth number *z*, pressure angle α, helix angle *β*); (2) Selection of the CAD modeling process using SolidWorks^®^ (SW) software (Toolbox Library); (3) Conversion of the CAD models of the gears into STL files (SW); (4) Selection of the polymeric materials for printing (PLA and PA66); (5) Selection of the 3D printing parameters for the chosen materials; (6) A slicing process using slicing software (BCN3D Cura 3.2); (7) Preparation of the printer (BCN3D Sigma 19) and transfer of the G-code files to it; (8) Gear printing; (9) Verification of the 3D-printed gears using conventional methods and various instruments; (10) Verification of the 3D-printed gears using laser scanning; and (11) Discussion and conclusions of the results.

The specific methods that were used are explained in detail in the next subsections.

### 2.1. The Helical Gear and Its Basic Parameters

Helical gears were selected in this work because of their ability to transmit power smoothly, quietly, and efficiently. Their applications include (1) the automotive industry, in transmission and differential systems; (2) industrial machinery, where they are commonly used in conveyor systems, compressors, pumps, and turbines; and (3) robotics, where they are essential for robotic arms and actuators, providing smooth and precise motion for accurate positioning and control.

A simple helical gear geometry is used, which is similar to the one that was used in the authors’ previous work [1], except for the tooth inclination parameter (*β*). In addition, the gears were printed using the same conditions that were previously defined.

Three types of helical gear models were designed with the same module (*m* = 4 mm), number of teeth (*z* = 17), and pressure angle (α = 20°), but different helix angles (*β*): (a) Model 1, *β* = 15°; (b) Model 2, *β* = 25°; and (c) Model 3, *β* = 35°. The selected module and number of teeth ensure a correct tooth profile for standard gears [10]. The typical range of *β* values in 3D-printed gears is between 15° and 35° [11].

In helical gears, the teeth generatrices are arranged in the form of a helix on the axoide cylinders, and are normally generated using the same tools and machines as those used to manufacture spur gears, in this case by tilting the cutting tool a certain angle *β*—the helix angle—with respect to the axoide cylinder generatrices (Figure 5a). Due to the tool’s inclination angle *β*, the transverse section of helical cylindrical gears does not correspond to the reference profile (normal profile), but rather conforms to the tool’s transverse profile (Figure 5). Thus, in helical gears there exist (a) Transverse section parameters (measured in the plane perpendicular to the gear axis), which correspond to the tool’s transverse profile and are denoted with subscript *t* (e.g., transverse pitch $p_{t}$) and (b) Normal section parameters (measured in the plane perpendicular to the teeth), which correspond to the tool’s normal profile and are denoted with subscript *n* (e.g., normal pitch $p_{n}$). In this work, only the intrinsic parameters with normal reference are used, as they do not depend on two meshed gears, since each gear is studied in isolation [29,30,31].

The geometry of helical gears is defined from a set of parameters called generation parameters, whose origin is in the standardized tools according to ISO 53-1998 [32], which are the number of teeth *z*, the normalized module $m_{n}$ of the reference profile, the pressure angle $\alpha_{n}$ of the tool, and the displacement coefficient *χ* (in this work, *χ* = 0, the case of normal gears for general mechanics [32]). The above parameters are common to spur gears, although for helical gears the helix angle *β* is added, which influences the generation (also called reference or pitch) diameter *d*, as shown in Table 1. The other geometric parameters (Figure 5b) are obtained by applying the formulas that are used in the initial generation parameters. They are as follows: *p*_n_ is the normal pitch, *d* is the reference diameter, $d_{a}$ is the tip diameter, $d_{b}$ is the base diameter, $d_{f}$ is the root diameter, *h* is the tooth depth, $h_{a}$ is the addendum, $h_{f}$ is the dedendum, *s*_1n_ is the normal tooth thickness, *s*_2n_ is the normal space width, and $r_{b}$ is the fillet radius [31]. Table 1 shows the values of these parameters for the three helical gear models presented in this work.

### 2.2. CAD Modeling Process Using SolidWorks

In this study, SolidWorks^®^ CAD software and its Toolbox library were used to model the gears, enabling parametric design of standardized mechanical elements according to ISO standards. Helical gears were generated by specifying the following parameters: normal module $m_{n}$ = 4 mm, number of teeth *z* = 17, normal pressure angle $\alpha_{n}$ = 20°, tooth width *b* = 20 mm, hub type A, hub inner diameter *d*_ih_ = 25 mm, and helix angle *β*. Figure 6 shows the three helical gear models (Model 1: *β* = 15°; Model 2: *β* = 25°; Model 3: *β* = 35°), with key parameters highlighted for Model 1. Detailed parameter values for each model are presented in Table 1. These CAD models served as the basis for 3D-printing the helical gears.

### 2.3. Conversion of the CAD Model of the Gear to an STL File

Additive manufacturing systems require 3D CAD models in compatible formats for subsequent processing in slicer software. The most common formats include STL (Stereolithography), SLC (Sliced Layer Contour), and HPGL (Hewlett-Packard Graphics Language). Most CAD packages (AutoCAD, CATIA, SolidEdge, SolidWorks, etc.) support STL export, which approximates surface geometry using triangular meshes containing only geometric data. In this study, STL files were generated from the gear CAD models using SolidWorks^®^2025 [8,9].

### 2.4. Selection of Printing Materials and Printing Parameters: Printed Models

To complement the previous study by Buj and Zayas [1], the same polymeric materials—PA66 and PLA—were employed, as they are widely used in the additive manufacturing of gears [1,6,10,14]. Specifically, Colorfabb PLA/PHA white filament and BASF Ultrafuse Nylon PA6/66 natural filament were used, both with nominal diameter 2.85 mm. The main characteristics of the filaments are found in Table 2.

Table 3 summarizes the 3D printing parameters used for both materials. To prevent hydrolytic degradation during thermal extrusion and to minimize internal void formation induced by moisture volatilization, the PA66 filament spools underwent a thermal dehydration pre-treatment in a forced-air convection oven at 80 °C for 12 h prior to printing. Following the pre-drying process, the spools were immediately transferred to a sealed dry-box feeding enclosure, maintaining a relative humidity below 10% before dimensional measurement. A 0.4 mm nozzle diameter was used for both materials. Three shells were defined (approximately 1.2 mm total wall thickness), with six top and bottom layers (approximately 1.2 mm total height). Figure 7 shows representative photographs of two helical gear specimens fabricated from PA66 (Figure 7a) and PLA (Figure 7b). Three replicates of each gear model were 3D-printed.

### 2.5. Dimensional Measurements with Conventional Instruments

This section describes the dimensional measurement procedures and the instruments used to measure the 3D-printed gears. The measured parameters include tooth thickness, sector span, and the diametral distance measurement for helical gears with an odd number of teeth.

#### 2.5.1. Tooth Thickness Measurement by the Tip Circle Method Using a Gear Tooth Vernier Caliper

The measurement method based on the tip circle consists of determining the chordal tooth thickness on the pitch cylinder of diameter d using an Alpa gear tooth vernier caliper, with a resolution of 0.01 mm. In this approach, the caliper is initially set to the theoretical tooth depth, $\bar{h_{a}}$, and the tooth thickness is measured. Figure 8a shows the measurement schematic, while Figure 8b illustrates its application on a PLA-printed helical gear.

Prior to the measurement process, the chordal height $\bar{h_{a}}$ and the theoretical chordal thickness of the tooth $\bar{s_{1}}$ are calculated using Equations (1)–(3), in which the parameters z, $m_{n}$, $\alpha_{n}$, $h_{a}$, $d_{a},$ and b have been previously defined.

(1) $$\bar{h_{a}}=\frac{Z\cdot m_{n}}{2}\cdot \left[1-\cos\left(\frac{\pi }{2\cdot Z}+\frac{2\cdot x_{n}\cdot \tan\alpha_{n}}{Z}\right)\right]+h_{a}$$

(2) $$h_{a}=\frac{d_{e}-d}{2}$$

(3) $$\bar{s_{1}}=Z\cdot m_{n}\cdot \sin\left(\frac{\pi }{2\cdot Z}+\frac{2\cdot x_{n}\cdot \tan\alpha_{n}}{Z}\right)$$

Table 4 contains the geometrical parameters used for the design of helical gears.

The procedure for measuring the tooth thickness is as follows (Figure 8): with the chordal height $\bar{h_{a}}$ on the instrument, the gear tooth vernier caliper is positioned in the normal plane of the tooth, approximately in the middle of the gear width b. Proper tangential contact of the probes of the instrument with the side faces of the tooth must be ensured (using the tooth thickness slider for this purpose) and then the vernier is fixed to minimize errors when measuring thickness $\bar{s_{1}}$. To facilitate the measurement process, the 3D-printed gear was secured in a vertical position using a bench vise. Measurements were carried out at several angular positions around the gear circumference, and the mean value for each gear was calculated. The results are presented in Section 3.

#### 2.5.2. Tooth Thickness Measurement by the Method of Measurement for Sector Span *W* Using a Disc Micrometer

The sector span (*W*) is measured by placing the parallel flat measuring faces of a gear tooth micrometer against the flanks of two teeth separated by a specified number of teeth and measuring the corresponding distance (*W*) (Figure 9). The number of teeth encompassed between the measuring faces is commonly referred to as the sector span of teeth, $z_{m}$. Measurements are typically performed at several angular positions around the gear circumference, and the mean value is calculated to account for pitch and profile deviations.

Figure 9a shows a schematic top view of a helical gear (width b, helix angle *β*), indicating the sector span W and disc micrometer placement. For helical gears, the micrometer should be positioned perpendicular to the tooth flanks and centered properly across measurements. Figure 9b shows the measurement process using an INPRESA analog disc micrometer (25–50 mm range, 0.01 mm accuracy) on a printed helical gear model.

For standard helical gears, the sector span W is calculated using Equation (4):

(4) $$W=m_{n}\cdot \cos\left(\alpha_{n}\right)\left[\pi \left(z_{m}-0.5\right)+z\cdot inv\left(\alpha_{t}\right)\right]$$

where the sector span of teeth is given by Equation (5):

(5) $$z_{m}=\frac{\alpha_{n}\cdot z_{v}}{180}\;+\;0.5$$

The sector span of teeth $z_{m}$ is approximated using the nearest integer value. The virtual number of teeth $z_{v}$ is calculated using Equation (6):

(6) $$z_{v}=\frac{Z}{\cos^{3}\left(\beta \right)}$$

Transverse pressure angle $\alpha_{t}$ is calculated by Equation (7):

(7) $$\alpha_{t}=\tan^{-1}\left(\frac{\tan\alpha_{n}}{\cos\beta }\right)$$

The involute function for $\alpha_{t}$ is given by Equation (8):

(8) $$inv\left(\alpha_{t}\right)=\tan\alpha_{t}-\alpha_{t}$$

Finally, for helical gears, the minimum face width $b_{min}$ required for accurate measurement of the sector span W is determined by Equation (9):

(9) $$b_{min}=W\cdot \sin\beta$$

The face width b (Figure 9a) of the helical gear should be verified to ensure that it satisfies the condition $b>b_{min}$; otherwise, one of the measurement elements may lose contact with the tooth flank, making the measurement unfeasible (Figure 10). Therefore, prior to the measurement process, the minimum face width $b_{min}$, the sector span $z_{m}$ (i.e., the number of teeth positioned between the micrometer discs), and the theoretical value of the sector span W were calculated using Equations (4)–(9). These theoretical values were subsequently used for comparison with the corresponding experimental measurements. Table 5 summarizes the calculated values of $z_{m}$, W, and $b_{min}$ for the three gear models analyzed in this study.

Since actual b = 20 mm is lower than $b_{min}$= 24.67 mm for Model 3, sector span was not measured in this case.

#### 2.5.3. Tooth Thickness Measurement by the Over-Rollers Measurement Method

This method is based on the use of calibrated rollers and is particularly suitable for helical gears or gears with non-standard pressure angles, for both external and internal gear teeth [31,34]. In the case of externally toothed gears, as considered in this study, calibrated rollers are positioned in opposing tooth spaces along the gear periphery (Figure 11). This allows the measurement of the diametral distance $d_{m}$ between the tangential contact points of two opposing rollers. This distance $d_{m}$ differs depending on whether the gear has an odd or an even number of teeth (Figure 11).

In this method, the diameter of the rollers, $d_{p}$, is selected based on the normal module $m_{n}$ and the normal pressure angle $\alpha_{n}$ of the gear. This selection is made by first calculating the ideal roller diameter, ${d^{\prime }}_{p}$, and then choosing a standard value that most closely approximates it [31,34].

For the calculation of the ideal roller diameter, ${d^{\prime }}_{p}$, in the case of externally toothed helical gears, Equation (10) is used [34]:

(10) $${d^{\prime }}_{p}=z_{v}\cdot m_{n}\cdot \cos\alpha_{n}\cdot \left(inv\left(\varphi_{v}\right)+\eta_{v}\right)$$

where the half-angle spacing $\eta_{v}$ is calculated according to Equation (11):

(11) $$\eta_{v}=\left(\frac{\pi }{2\cdot \alpha_{n}}-inv\left(\alpha_{n}\right)\right)-\frac{2\cdot \chi_{n}\cdot \tan\alpha_{n}}{z_{v}}$$

With the pressure angle ${\alpha^{\prime }}_{v}$ at the point where the roller is tangent to the tooth surface Equation (12):

(12) $${\alpha^{\prime }}_{v}=\cos^{-1}\left(\frac{z_{v}\cdot \cos\left(\alpha_{n}\right)}{z_{v}+2\cdot \chi_{n}}\right)$$

where $\varphi_{v}$ is the pressure angle at the center of the rod, determined according to Equation (13):

(13) $$\varphi_{v}=\tan\alpha_{n}+\eta_{v}$$

For each helical gear with a different helix angle *β*, a specific roller diameter must be selected, as each model yields a distinct value of the ideal roller diameter, ${d^{\prime }}_{p}$, as summarized in Table 6.

In this work, the rollers available in the Manufacturing Technologies Laboratory of the Department of Mechanical Engineering (DEM) at ETSEIB, with a diameter of $d_{p}$ = 9 mm, were used. Although this value differs from the ideal diameter, ${d^{\prime }}_{p}$, the measurements remain feasible, as demonstrated below. Once the roller diameter $d_{p}$ is selected, preliminary calculations are required to determine the theoretical values of the diametral distance $d_{m}$ between the tangents to two opposing rollers [31].

The diametral distance $d_{m}$ for gears with an odd number of teeth, as in the case of the present models (*z* = 17), is calculated using Equation (14), where the parameters *z*, $m_{n}$, $\alpha_{t}$, $\varphi_{v}$, *β*, and $d_{p}$ have been previously defined.

(14) $$d_{m}=\frac{z\cdot m_{n}\cdot \cos\left(\alpha_{t}\right)}{\cos\left(\beta \right)\cdot \cos\left(\varphi_{v}\right)}\;\cdot \cos\frac{90{}^{\circ}}{z}+d_{p}$$

For each helical gear model, the theoretical diametral distance $d_{m}$ is obtained, as summarized in Table 7. These values serve as reference data and are compared with the corresponding experimentally measured values, $d_{mreal}$.

The measurement of helical gears using rollers is a complex procedure that typically requires two operators to ensure accuracy. The methodology involves securing the gear in a bench vise. One operator positions the two rollers in opposite horizontal locations, ensuring tangential contact with the flanks of the helical teeth, as shown in Figure 12, while the second operator measures the resulting diametral distance $d_{mreal}$ using a TESA digital caliper with a measuring range of 0–150 mm and 0.01 mm resolution. The measurement results for $d_{mreal}$ are presented in Section 3.

### 2.6. Dimensional Measurements Using Laser Scanning

The geometry of the printed helical gear models was evaluated using a ZEISS T-SCAN Hawk 2 3D laser scanner (Oberkochen, Germany) to determine dimensional deviations. Data acquisition and initial processing were performed using the basic version of ZEISS INSPECT software, followed by further quantitative analysis using CloudCompare, an open-source 3D inspection software. The scanner has a volumetric accuracy of 0.020 mm + 0.015 mm/m and a spatial resolution of 0.2 mm.

The scanning procedure is illustrated in Figure 13 and comprises the following stages.

(1) Preparation: The scanner was calibrated using the calibration artifact supplied with the equipment (Figure 13a). Reference targets were then placed on both sides of the printed gear to clearly distinguish between its two faces (Figure 13b).

(2) Scanning and data processing: Each gear was digitized in three sections, which were subsequently registered and merged to reconstruct its complete geometry. For the first scan, the gear was placed horizontally on the rotary scanning platform (Figure 13c). The reference points were scanned by varying the distance and orientation of the scanner head relative to the part. The lower plane in the Z-direction, located 20 mm above the base, was then selected in the software, and the part was scanned. The surfaces of interest were subsequently selected, and any scanned data not belonging to the workpiece was removed (Figure 13d,e).

For the second scan, the gear was placed vertically on the scanning platform (Figure 13f), and the procedure described above was repeated to capture the corresponding surface (Figure 13g). The Transform Measurement Series function was then used to merge the first and second scans. Only the common reference points located on the faces of the part were used for alignment and merging (Figure 13h).

For the third scan, the vertically oriented gear was rotated by 120° relative to its previous position, and the same procedure was repeated. Once the entire part had been scanned, the Finish Scanning and Polygonize function was selected. Standard smoothing was subsequently applied to reduce minor surface imperfections caused by noise. Any remaining holes in the scanned surface were then automatically or interactively filled.

(3) Feature identification: The STEP file of the nominal part was imported into the software (Figure 13i). The measured surface was then aligned with the nominal surface using an automatic best-fit procedure (Figure 13j).

(4) Deviation analysis: Finally, the Compare Surfaces function was selected to generate the deviation map (Figure 13k).

The measurement results obtained from the scanner and analyzed using ZEISS INSPECT are presented in the Results and Discussion section.

Figure 14 illustrates the procedure used in CloudCompare to generate the deviation map and calculate the distance statistics for the comparison between the nominal CAD model and the scanned model. The procedure comprises the following steps: (1) obtaining the reference mesh from the CAD model designed in SolidWorks^®^ and the scanned mesh generated using ZEISS INSPECT, as described previously; (2) importing both meshes in STL format into CloudCompare; (3) performing an initial alignment using the Align (point pairs picking) tool; (4) performing fine registration using the Iterative Closest Point (ICP) algorithm; (5) verifying the final alignment between the CAD and scanned models; (6) calculating the distances using the Cloud-to-Mesh distance tool, with the scanned mesh represented by a point cloud and the CAD mesh used as the reference surface; (7) generating the deviation map to visualize the spatial distribution of the deviations; and (8) calculating the distance statistics using the Compute statistical parameters function, including the mean distance, standard deviation, root mean square (RMS) distance, and minimum and maximum distances.

The measurement results obtained from the CloudCompare software are presented in the Section 3.

## 3. Results and Discussion

### 3.1. Conventional Measurement Methods

This section presents the results of the tooth thickness ($s_{1real}$), sector span ($W_{real}$), and diametral distance ($d_{mreal}$) measurements, obtained using conventional measurement methods. A summarized version of these results was presented at the CNIM 2025 conference [35], and the results were supplemented with additional laser scanner measurements (see Section 3.2).

#### 3.1.1. Tooth Thickness $s_{1real}$

As previously explained in Section 2.4, for each helical gear model and material, three replicates were 3D-printed. For each specimen, three measurements were performed on different teeth to assess variability. Table 8 contains the mean thickness values for each gear, as well as the mean, standard deviation (STD), confidence intervals, theoretical value, and relative error for each group.

Two-way ANOVA was carried out with 95% confidence, and *p*-values of 0.040, 0.473, and 0.3122 were obtained for the material, the angle, and the interaction, respectively. In this case, only the effect of the material is statistically significant.

Lower error was found for PLA than for PA66 when *β* = 15° and *β* = 25° are considered. On the contrary, lower error was found for PA66 than for PLA when *β* = 35° is selected. This corresponds to lower absolute thickness values for PLA than for PA66, when the same angle is considered. This can be attributed to the fact that, since the shrinkage coefficient of PA66 is higher than that of PLA, actual teeth of 3D-printed PA66 parts will be shorter than actual teeth of 3D-printed PLA. When measuring thickness on PA66 parts, the position of the probes of the gear tooth vernier caliper will be lower than expected, and this corresponds to a higher thickness value. Thermal shrinkage of polyamides typically ranges between 0.7% and 3%, while it ranges between 0.3% and 0.5% for PLA [36].

Figure 15 illustrates the deviation of the mean measured value with respect to the theoretical chordal tooth thickness, $\bar{s_{1}}$.

Based on Table 8 and Figure 15, it can be concluded that, for the first two types of gears (Model 1 with *β* = 15° and Model 2 with *β* = 25°), PLA shows lower relative error than PA66. However, for Model 3 (*β* = 35°), PA66 gears exhibit lower relative error with respect to the theoretical value compared to the PLA specimens. Lowest relative error of 0.27% was obtained for PA66 and 15°, while highest relative error was found for PLA and 35°.

#### 3.1.2. Sector Span $W_{real}$

For Models 1 and 2, three sector span ${(W}_{real}$) measurements were performed across different toothed sectors of each 3D-printed helical gear. The results are presented in Table 9. Model 3 does not satisfy the condition $b>b_{min}$ (Table 5) and, therefore, sector span was not measured in this case.

When comparing the data for *β* = 15° for the two materials with a one-way ANOVA, a *p*-value of 0.00000652 is obtained. As for *β* = 25°, a *p*-value of 0.001526 is found. In both cases, there is statistical significance between the materials.

Figure 16 illustrates the measured chordal thickness *W* values, as well as its theoretical value.

The results in Table 9 and Figure 16 indicate that PA66 gears provided lower relative error than PLA ones. As explained in Section 3.1.2, PA66 exhibits a higher shrinkage coefficient than PLA. This leads to smaller PA66 3D-printed gears, with shorter chordal distance, which, in this case, is closer to the theoretical value.

#### 3.1.3. Diametral Distance $d_{mreal}$

The diametral distance ($d_{mreal}$) measurement procedure was repeated three times per 3D-printed helical gear on different tooth pairs to assess variability. Results are presented in Table 10 and Figure 17.

When comparing the data for *β* = 15° for the two materials with a one-way ANOVA, a *p*-value of 0.402 is obtained. As for *β* = 25°, a *p*-value of 0.498 is found. For *β* = 35°, the *p*-value was 0.323. In all cases, there is no evidence of statistical significance between materials.

Figure 17 shows the diametral distance values, as well as its theoretical value.

### 3.2. Laser Scanner Measurement

#### 3.2.1. Scanner Measurement by ZeissInpect Software

Figure 18 and Figure 19 show the scanner measurements obtained for replicates 1, 2, and 3 of the PLA specimens with *β* = 15° and *β* = 35°, respectively, using ZEISS INSPECT software. All three replicates exhibited similar behavior.

Figure 18 shows that, for PLA gears with *β* = 15°, most areas are shown in green, whereas the tooth root exhibits a material deficit, in blue, around 0.4 mm. This effect is only marginally observed for *β* = 35° (Figure 19). The material deficit may be related to the geometry of the fillet radius at the tooth root (Figure 5b). Although the standard specifies a fillet radius of 0.38·*m_n_*, where *m_n_* is the module (Table 1), the width of the space through which the diffuser nozzle must travel is smaller in a 15° helical gear than in a 35° helical gear.

In this case, most surfaces are green, showing similarities between the theoretical and measured surfaces.

Figure 20 and Figure 21 depict the scanner measurements obtained for replicates 1, 2, and 3 of the PA66 specimens with *β* = 15° and *β* = 35°, respectively, using ZEISS INSPECT software.

Figure 20 and Figure 21 show that, as a general trend, the teeth surfaces have some areas in blue, with a maximum material excess of approximately 0.5 mm, especially at the crests. This may be attributed to the fact that PA66 exhibits a higher shrinkage coefficient than PLA [35]. Furthermore, a yellowish hue is observed on the front face and/or on the bore internal surface of some replicas, indicating a slight material excess of around 0.1 mm.

In summary, the PLA gears have a slight material deficit at the tooth root, while the PA66 gears show a material deficit mainly at the tooth crest.

#### 3.2.2. Deviation Map and Error Statistics

This section presents a combined analysis of the deviation maps and error statistics between the measured and the theoretical surfaces of the gears. It provides a comprehensive qualitative and quantitative assessment of the dimensional deviations of the 3D-printed helical gears.

Following the scanning procedure described in Section 2.6, the resulting meshes were exported from ZEISS INSPECT in STL format and imported into CloudCompare. The scanned meshes were then compared with the reference CAD mesh to obtain the corresponding deviation maps and extract the associated statistical data.

The comparison results are first presented for replicate 1 of the PLA gears with helix angles of 15° and 35° (Figure 22 and Figure 23). The corresponding results for replicate 1 of the PA66 gears with 15° and 35° are then presented using the same approach (Figure 24 and Figure 25).

Figure 22 (PLA specimen with *β* = 15°, replicate 1) shows that, from the initial alignment and the application of the Iterative Closest Point (ICP) algorithm, the relative position of the geometries being compared was optimized to improve their correspondence. This is evidenced by the decrease in the RMS (Root Mean Square) value from 0.199123 (after the initial alignment) to 0.15591 (after the second alignment). Figure 22a and Figure 22b show a screenshot of the first and the second alignment respectively. Figure 22c contains the comparison between the theoretical and the measured surfaces. It yields the following values: (1) the minimum distance is 0 mm, (2) the maximum distance is approximately 0.87 mm, (3) the approximate average distance is 0.11 mm, and (4) the approximate maximum error is 0.31 mm. Figure 22d shows the distribution of signed distances (C2M signed distances) and the fit of a Gaussian distribution, with an approximate mean of +0.216 mm and an approximate standard deviation of 0.156 mm.

Similarly, the analysis based on Figure 23 (PLA specimen with β = 35°, replicate 1) demonstrates that the geometric alignment was optimized, as the RMS value decreased from 0.309391 (initial alignment) to 0.0823645 (second alignment). Figure 23a and Figure 23b show a screenshot of the first and the second alignment, respectively. Figure 23c contains the comparison between the theoretical and the measured surfaces. Figure 23d displays the distribution of signed distances and the fit of a Gaussian distribution, showing a mean of approximately +0.10 mm and a standard deviation of approximately 0.086 mm. The calculation of distances between the compared surfaces yields the following results: (1) the minimum distance is 0 mm; (2) the maximum distance is approximately 1.02 mm; (3) the average distance is approximately 0.02 mm; and (4) the maximum error is approximately 0.36 mm. According to Figure 23d, the dimensions seem to be more uniformly distributed than for β = 15°. That can be understood as a more uniform geometry of this 3D-printed gear. In addition, the average distance is low, close to 0.

Figure 24 and Figure 25 below correspond to replicate 1 of the PA66 gears with helix angles of 15° and 35°, respectively.

Figure 24a shows a screenshot of the second alignment. Figure 24b contains the distances between the compared surfaces: (1) the minimum distance is 0 mm, (2) the maximum distance is approximately 1.94 mm, (3) the approximate average distance is 0.32 mm, and (4) the approximate maximum error is 0.31 mm. Figure 24c shows the distribution of signed distances and the fit of a Gaussian distribution, with an approximate mean of +0.37 mm and an approximate standard deviation of 0.41 mm.

Similarly, the analysis of the graphs and data depicted in Figure 25a (PA66 specimen with *β* = 35°, replicate 1) shows that the geometric alignment was optimized, as the RMS value decreased from 0.123661 (initial alignment) to 0.0823982 (second alignment). The calculation of distances between the compared surfaces in Figure 25b yields the following: (1) the minimum distance is 0 mm, (2) the maximum distance is approximately 0.72 mm, (3) the approximate average distance is 0.02 mm, and (4) the approximate maximum error is 0.36 mm. Figure 25c shows the distribution of signed distances and the fit of a Gaussian distribution, with an approximate average value of +0.10 mm and an approximate standard deviation of 0.086 mm.

## 4. Conclusions

In the present study, the dimensions of PLA and PA66 3D-printed helical gears were compared. Three gear models with different helix angles (*β* = 15°, 25°, and 35°) were fabricated, all with a module of 4 mm and 17 teeth.

The dimensional characteristics of the 3D-printed gears were evaluated using three measurement methods: (a) the tip-circle-based method, (b) the sector-span method, and (c) the over-rollers method. The dimensional parameters evaluated were the actual tooth thickness ($s_{1real}$), chordal distance ($W_{real}$), and diametral distance ($d_{mreal}$). The main conclusions are as follows:

- For PLA gears with *β* = 15° and *β* = 25°, tooth thickness ($s_{1real}$) measurements were slightly closer to the theoretical value ($\bar{s_{1}}$) than those for PA66. For gears with *β* = 35°, PA66 exhibited closer values than PLA. The difference between the two materials was statistically significant.
- The chordal distance ${(W}_{real}$) values of PA66 gears was closer to the theoretical value (W) than those of PLA gears. The difference between the two materials was statistically significant, for both *β* = 15° and *β* = 25°. For *β* = 35°, the chordal distance could not be measured, because the 3D parts were too thin.
- Regarding the measurement of the diametral distance ($d_{mreal}$), PLA gears showed lower dimensional deviations than PA66 gears. In addition, unlike the rest of the samples, for the *β* = 25° gears, measured values were lower than theoretical ones. This can be attributed to the complexity of the measurement methodology. On the other hand, these results indicate relatively low dimensional deviations but do not constitute an overall gear-accuracy assessment or demonstrate suitability for meshing or power-transmission applications, as functional gear performance and ISO 1328 gear-quality classification were not evaluated.

The comparison between the laser scanner measured surfaces and the theoretical ones leads to the following conclusions:

- For PLA gears, the measured and theoretical surfaces are generally in good agreement, except at the tooth root for *β* = 15°, where the 3D-printed parts exhibit a material deficit of approximately 0.4 mm in depth. The PA66 gears exhibit a deficit of material at the tooth crest, with a slight excess of material at the tooth frontal faces, for both *β* = 15° and *β* = 35°.
- From the deviation maps it can be concluded that average deviation obtained when *β* = 15° is higher than the average deviation for *β* = 35°, regardless of the material that is considered.

This work provides a combined methodology for gear dimensional measurement, including both the use of conventional metrology devices and laser scanner.

## Figures and Tables

**Figure 1:**
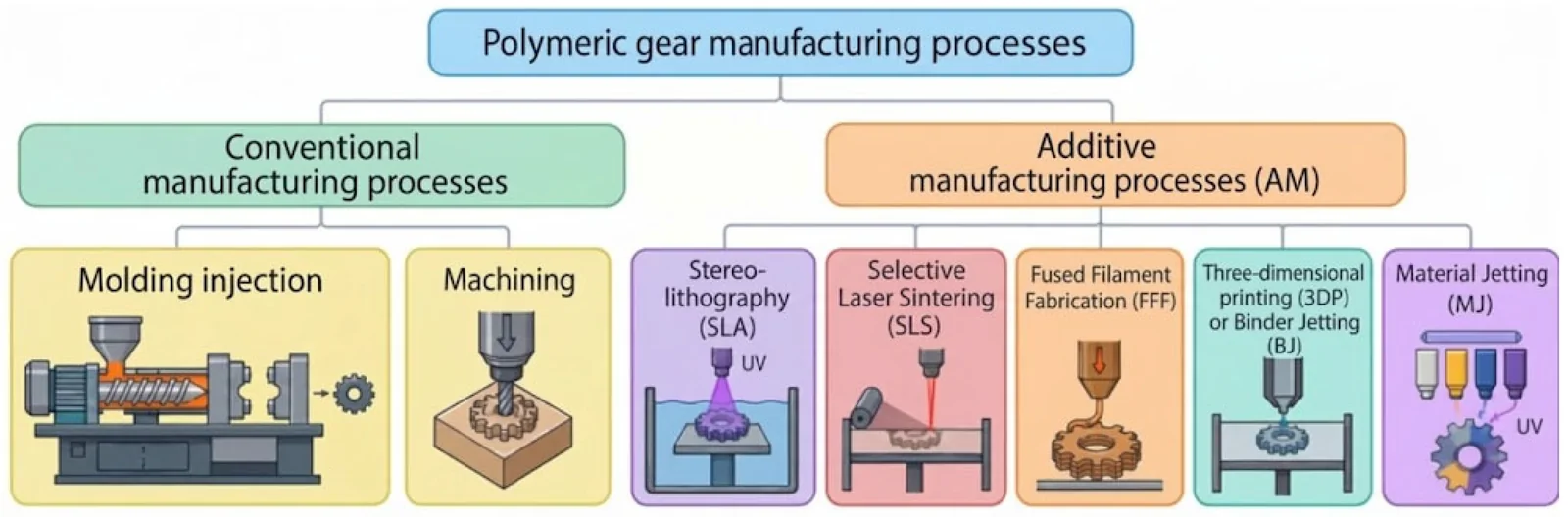
Classification of polymeric gear manufacturing processes.

**Figure 2:**
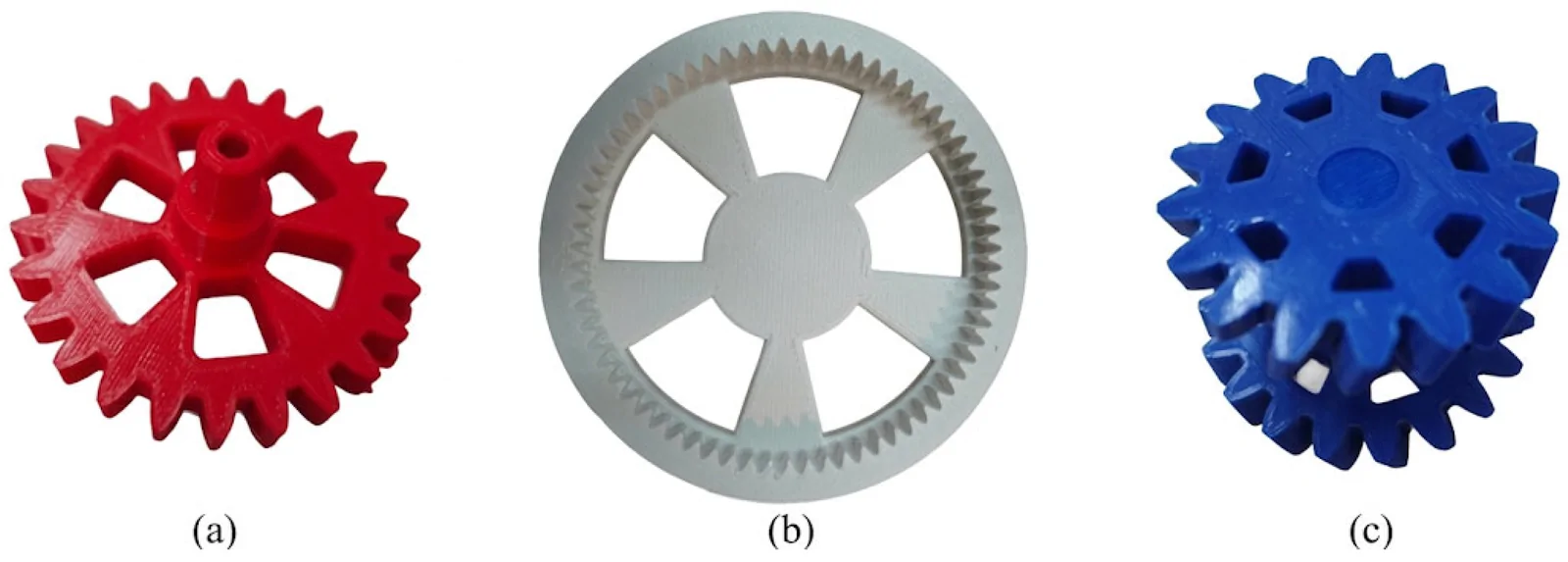
Example of polymer gears printed with different geometric shapes and colors: (**a**) red-colored spur gear, (**b**) white-colored inner gear, and (**c**) two planetary joint gears in blue color.

**Figure 3:**
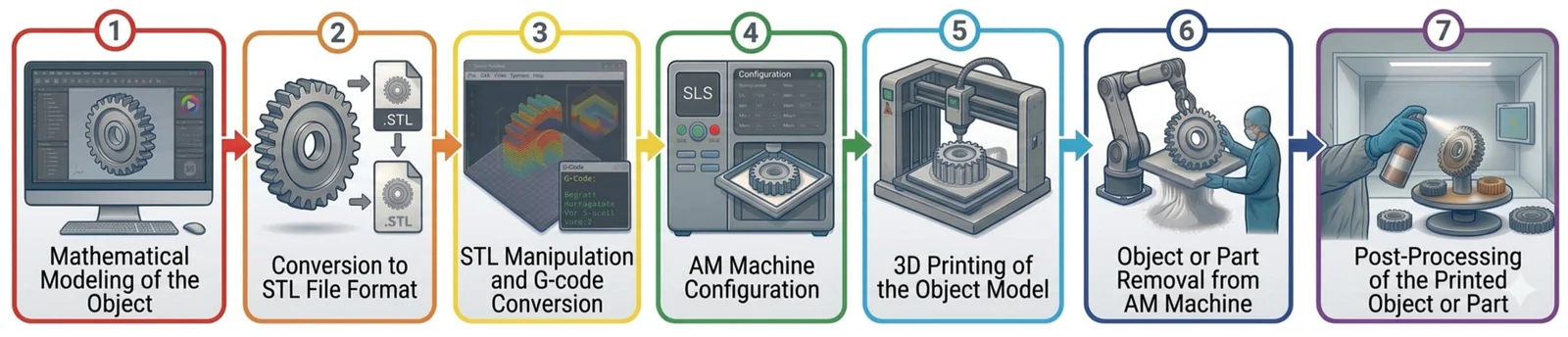
Common steps in additive manufacturing technologies.

**Figure 4:**
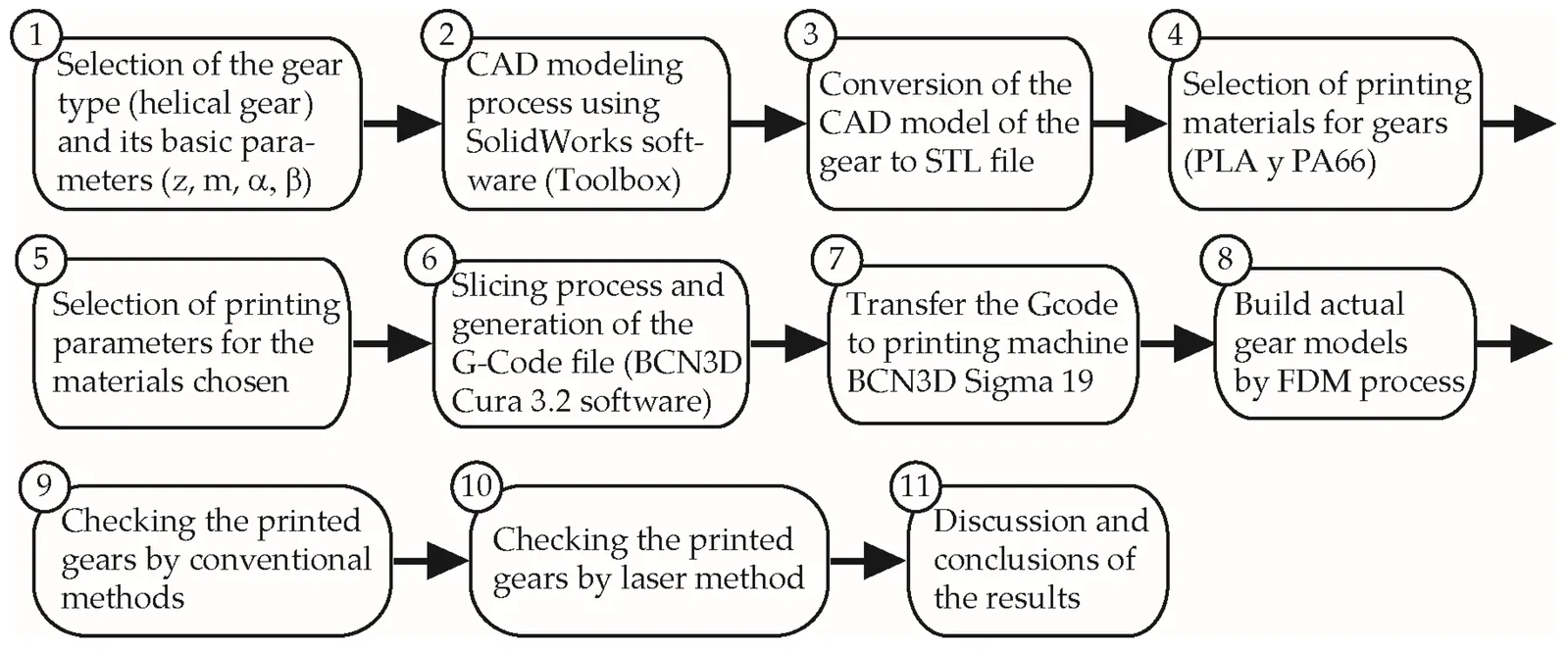
General methodology of the work.

**Figure 5:**
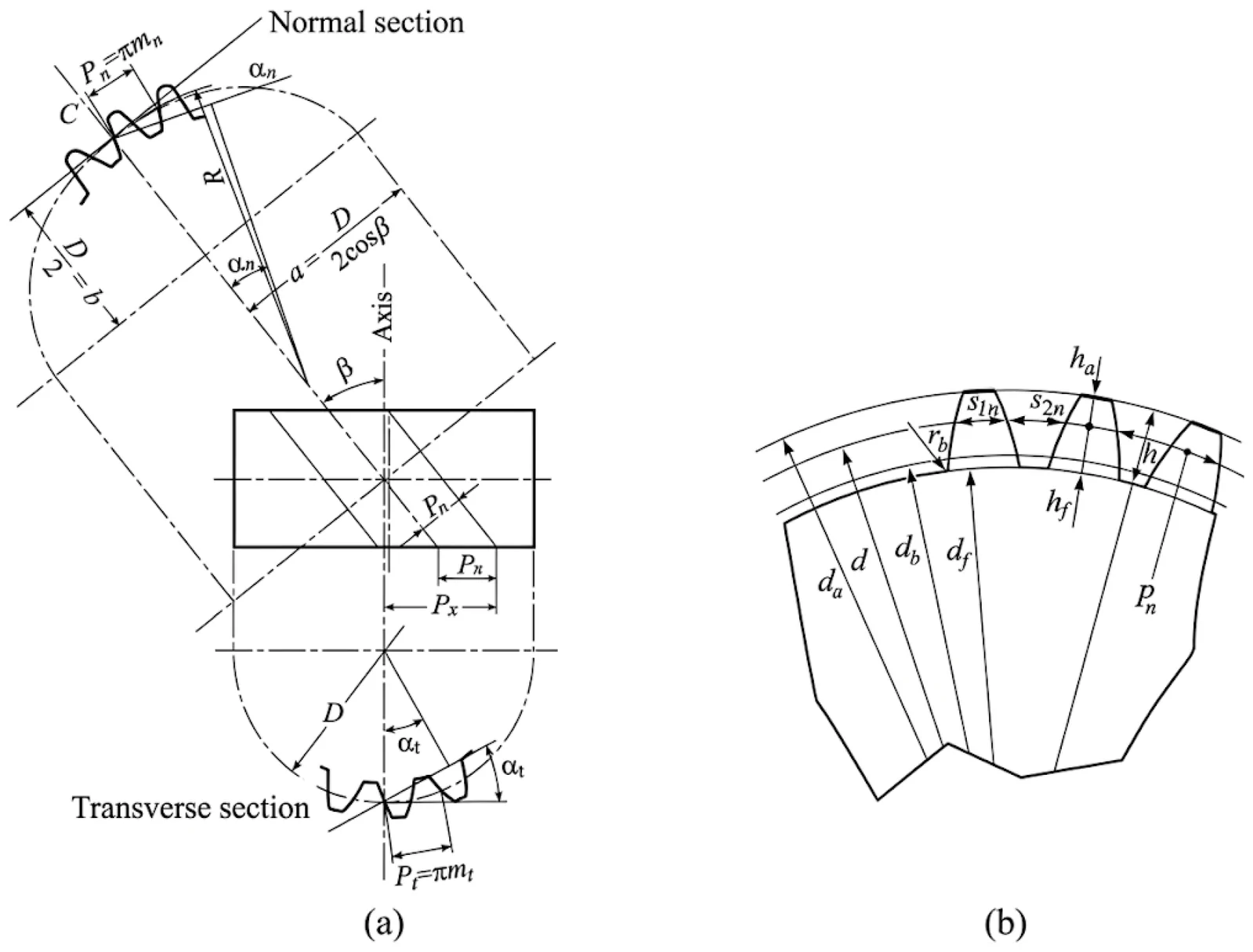
Geometrical parameters of a helical gear. (**a**) Normal and transverse sections. (**b**) Section of a gear with its basic parameters. Modified from sources [1,31].

**Figure 6:**
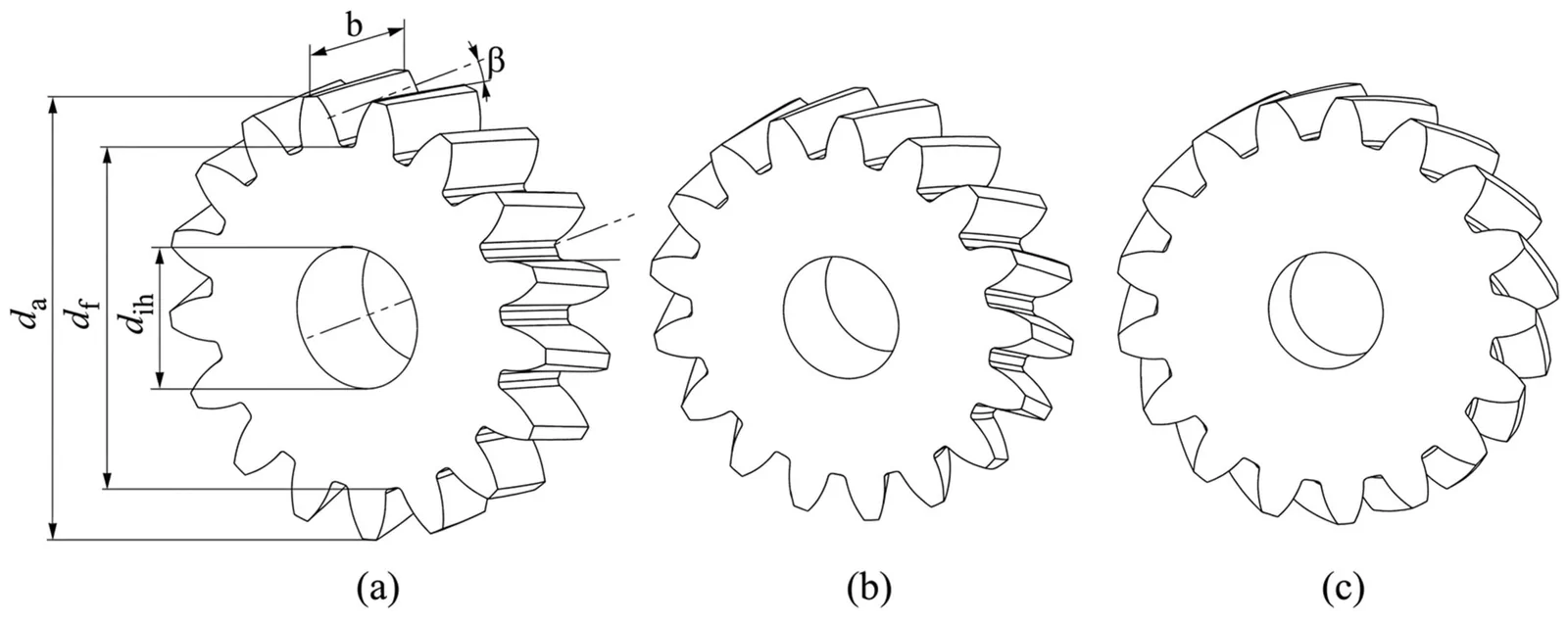
3D Models: (**a**) Model 1 (*β* = 15°), (**b**) Model 2 (*β* = 25°), and (**c**) Model 3 (*β* = 35°).

**Figure 7:**
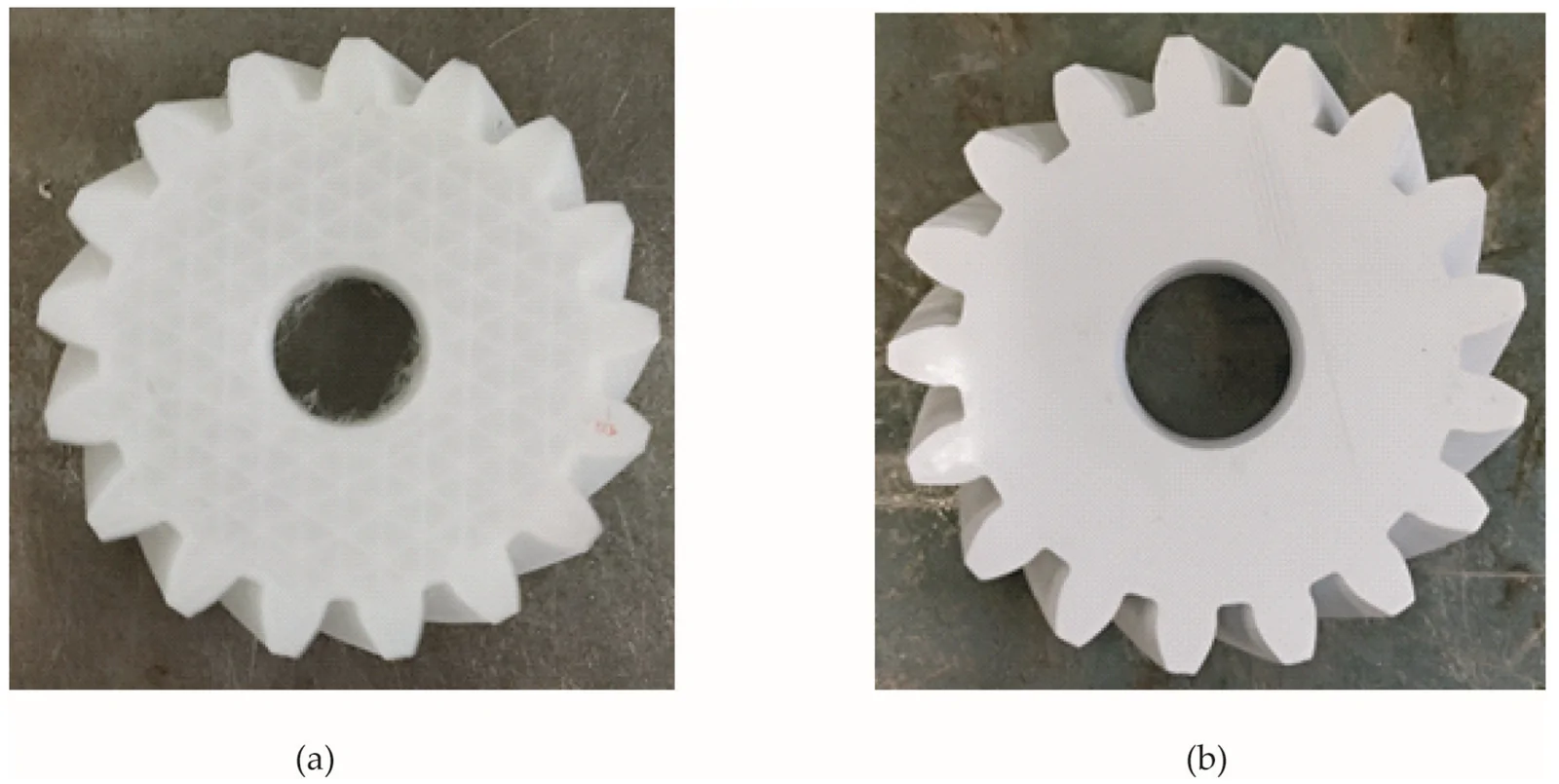
Images of two 3D-printed gears: (**a**) Model 1 fabricated in PA66; (**b**) Model 1 fabricated in PLA.

**Figure 8:**
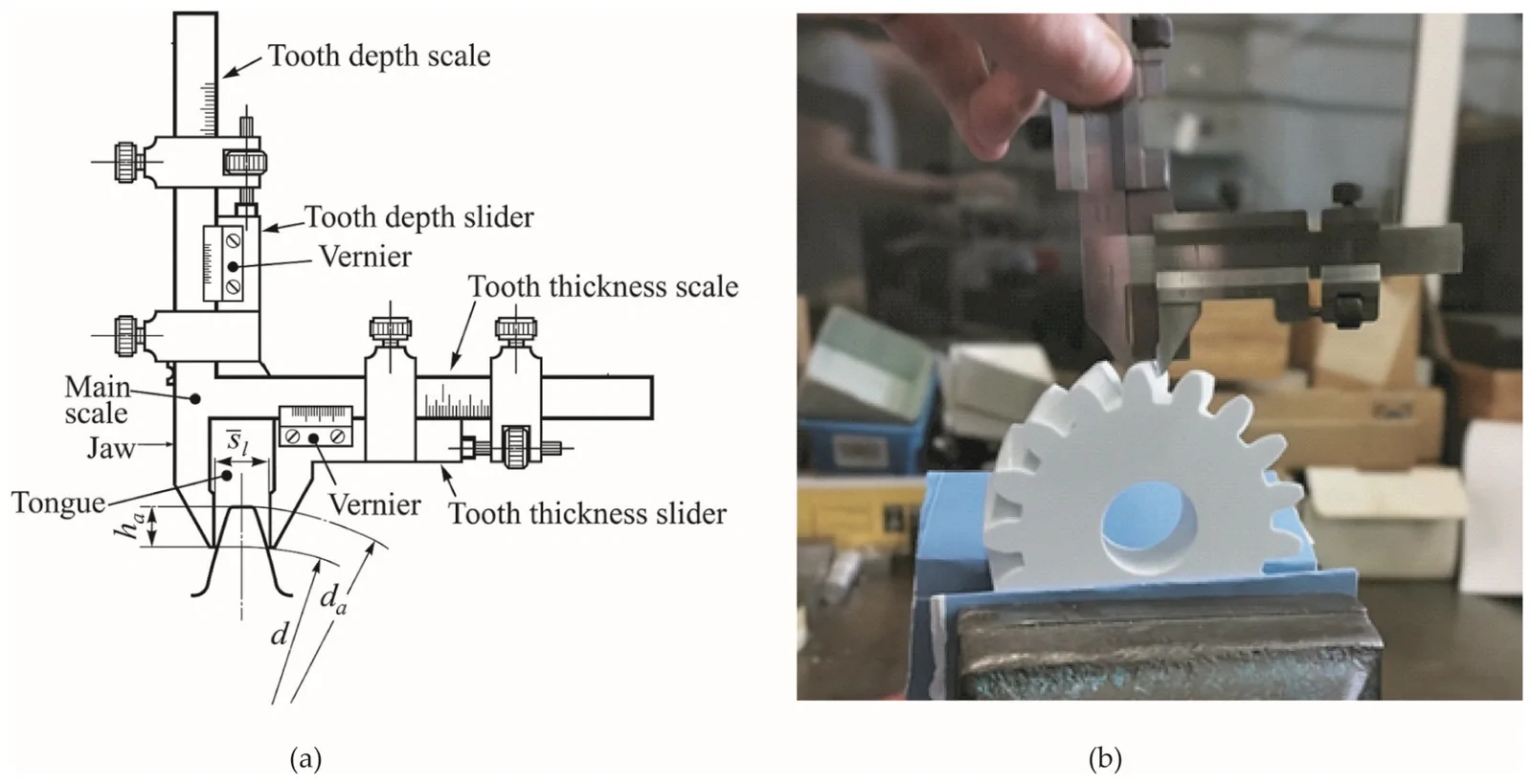
Measurement with gear tooth vernier caliper: (**a**) Parts of the instrument and required parameters ($\bar{h_{a}}$, $\bar{s_{1}}$) for measurement (modified from [31]); (**b**) Measuring the tooth thickness of a 3D-printed gear.

**Figure 9:**
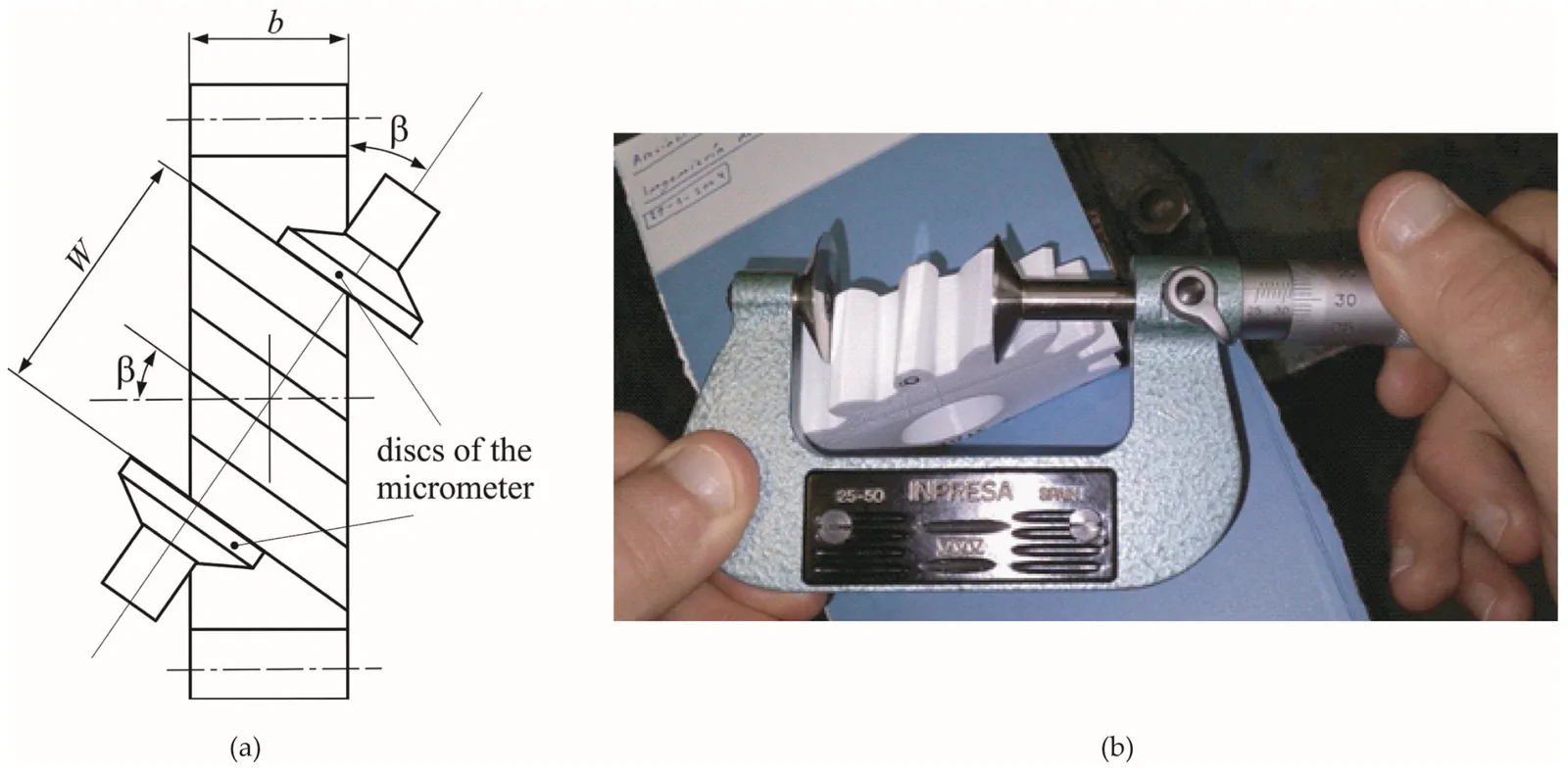
Disc micrometer measurement: (**a**) sector span *W* indication on helical gear; (**b**) measurement of distance *W* using an analog micrometer ($z_{m}$ = 3). Modified from [31,33].

**Figure 10:**
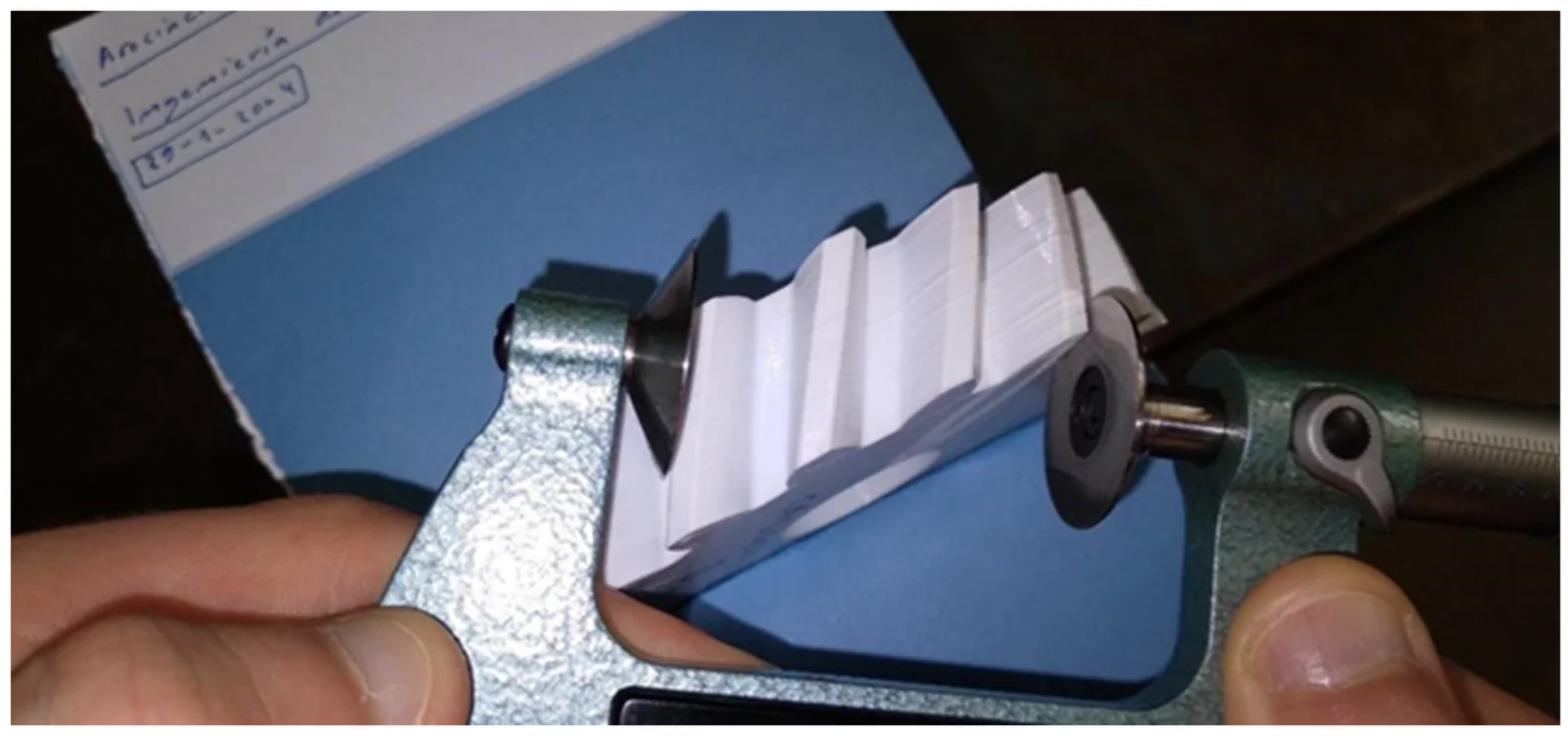
Incorrect positioning of the disc micrometer when measuring, due to insufficient width *b* of the printed model 3 (*β* = 35°, *b* = 20 mm). Modified from [33].

**Figure 11:**
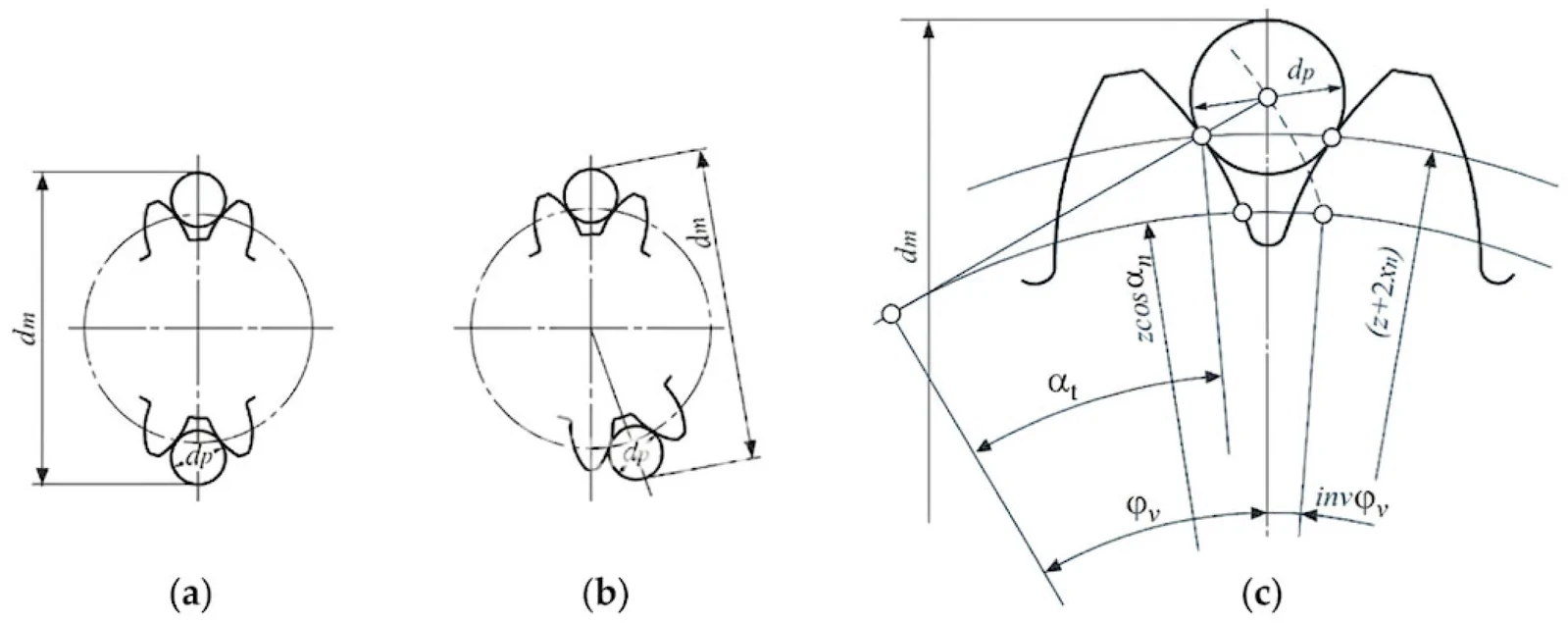
(**a**) $d_{m}$ in gears with even number of teeth, (**b**) $d_{m}$ in gears with odd number of teeth, (**c**) rollers dimension for helical gear (shown in axis section). Modified from [31,34].

**Figure 12:**
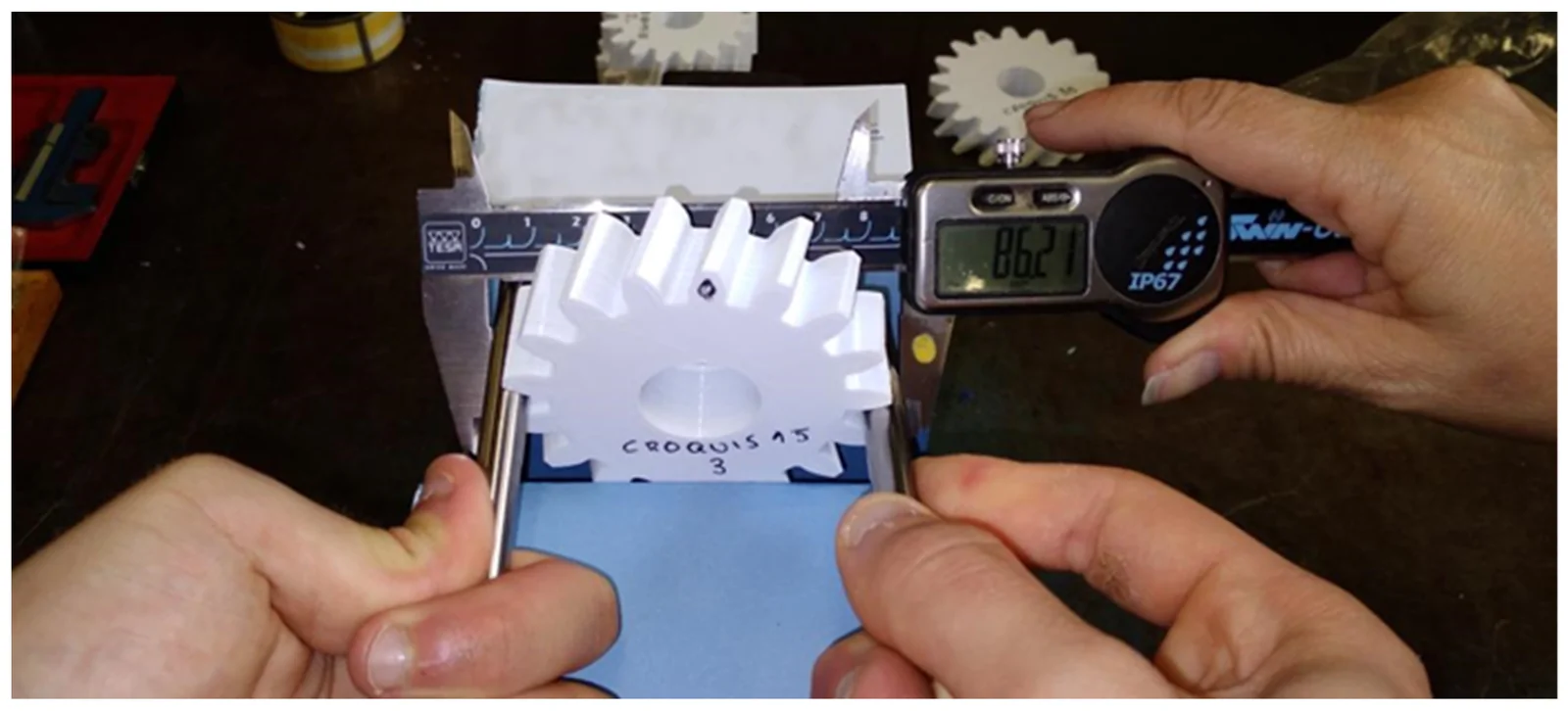
Diametral distance measurement on a printed helical gear using rods and a caliper. Modified from [33].

**Figure 13:**
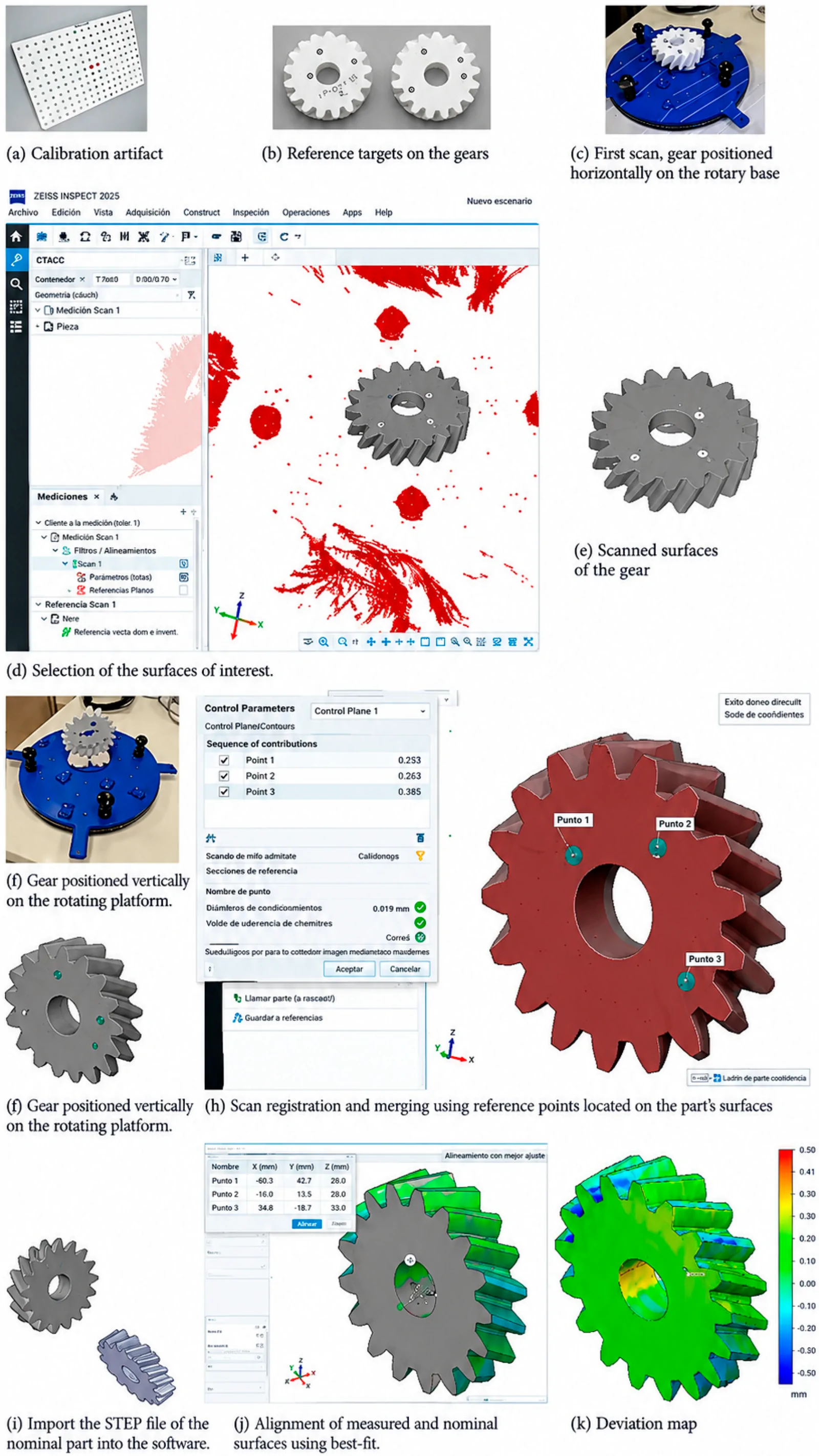
Laser scanning measurement procedure for 3D-printed helical gears.

**Figure 14:**
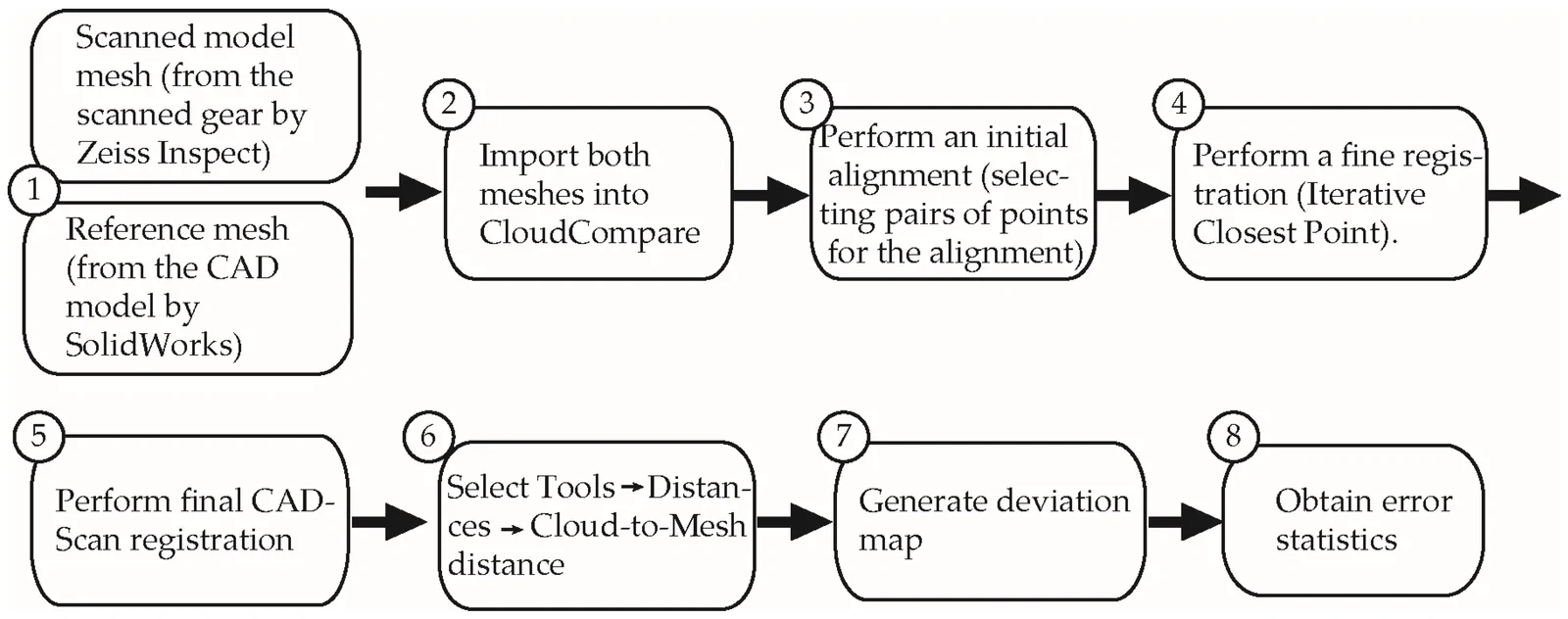
Procedure for obtaining the deviation map and error statistics using CloudCompare software.

**Figure 15:**
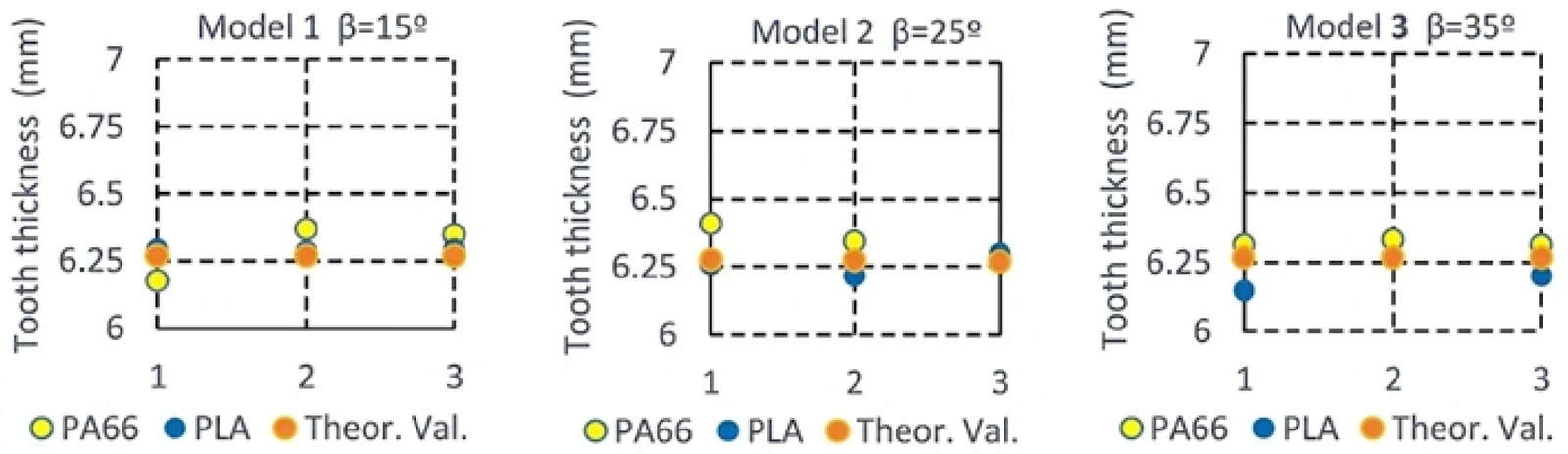
Variation of the mean value of tooth thickness for each model and material.

**Figure 16:**
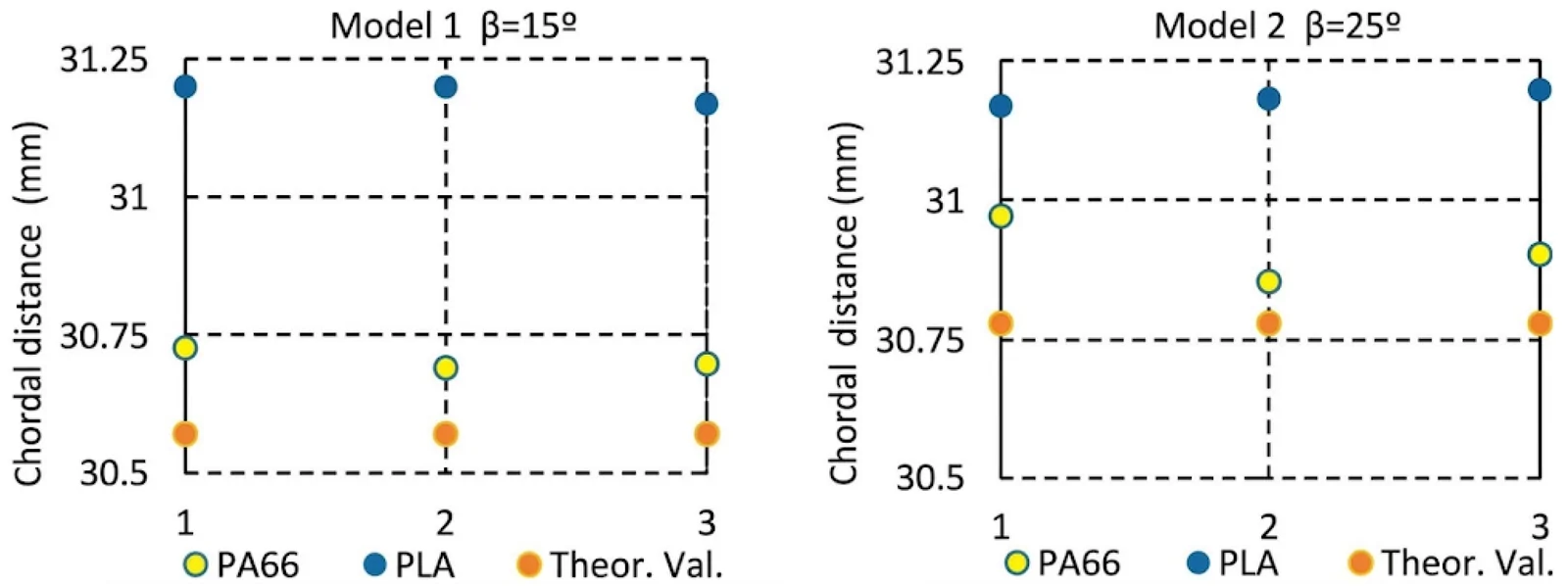
Variation of the mean chordal thickness for Models 1 and 2 as a function of material.

**Figure 17:**
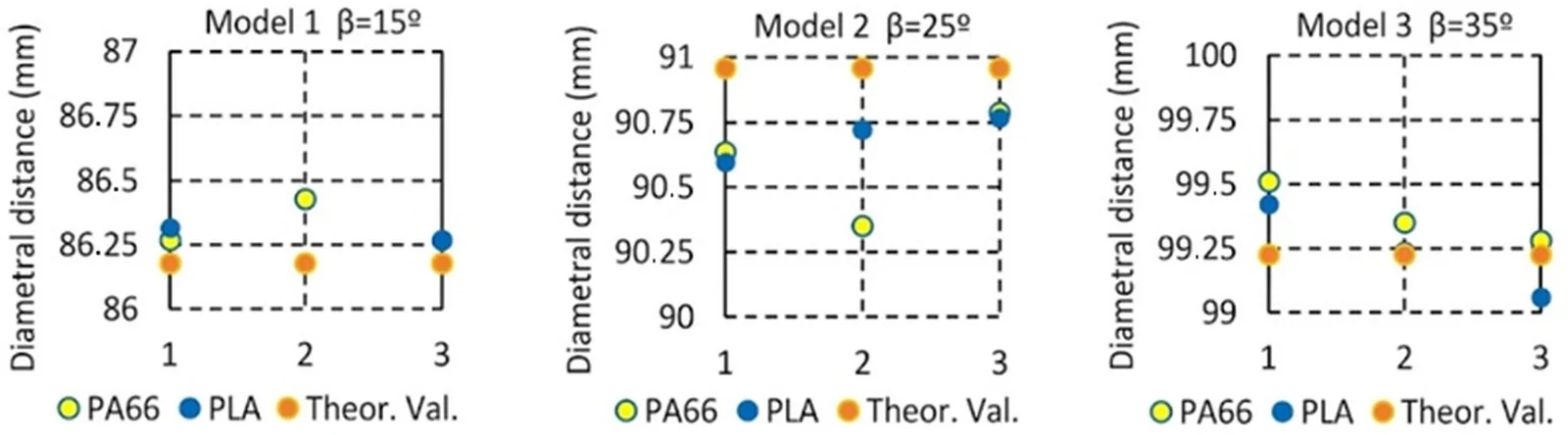
Mean diametral distance variation for each model and material.

**Figure 18:**
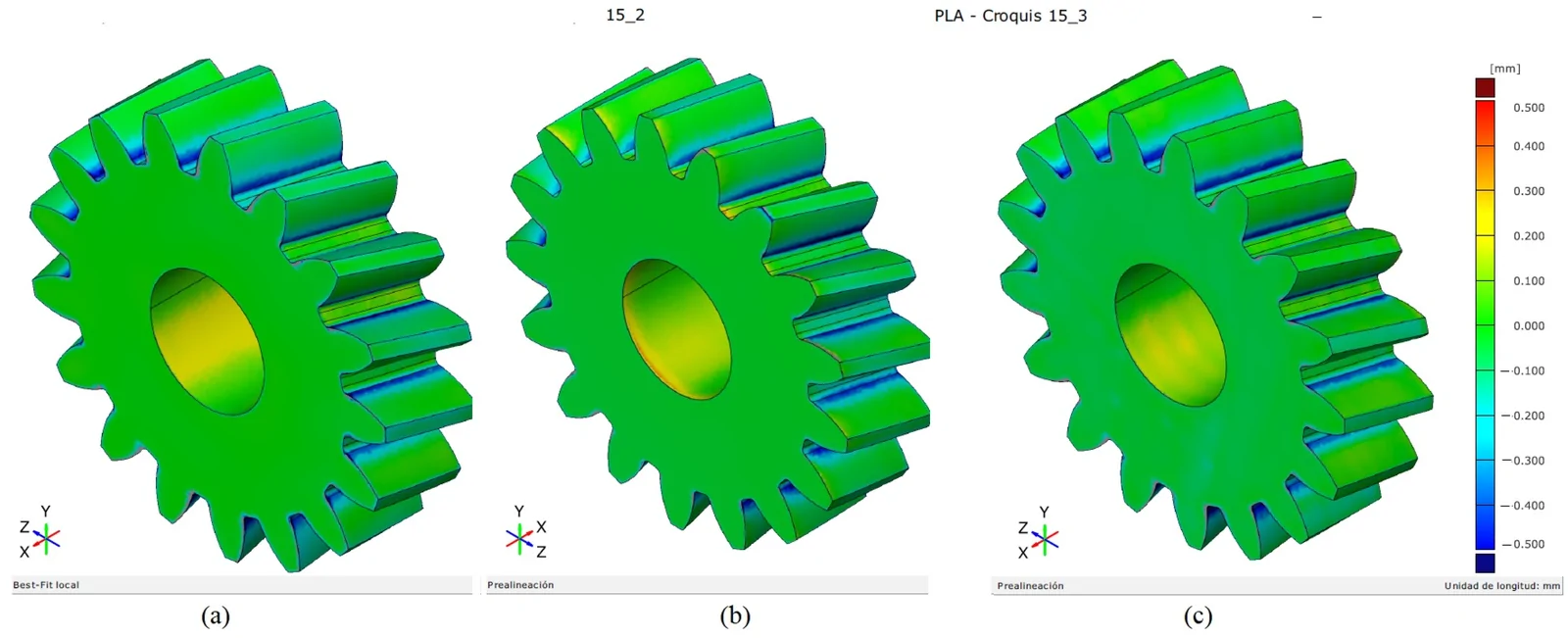
Scanned gear surfaces of PLA specimens with *β* = 15°: (**a**) replicate 1, (**b**) replicate 2, and (**c**) replicate 3, obtained using ZEISS INSPECT software.

**Figure 19:**
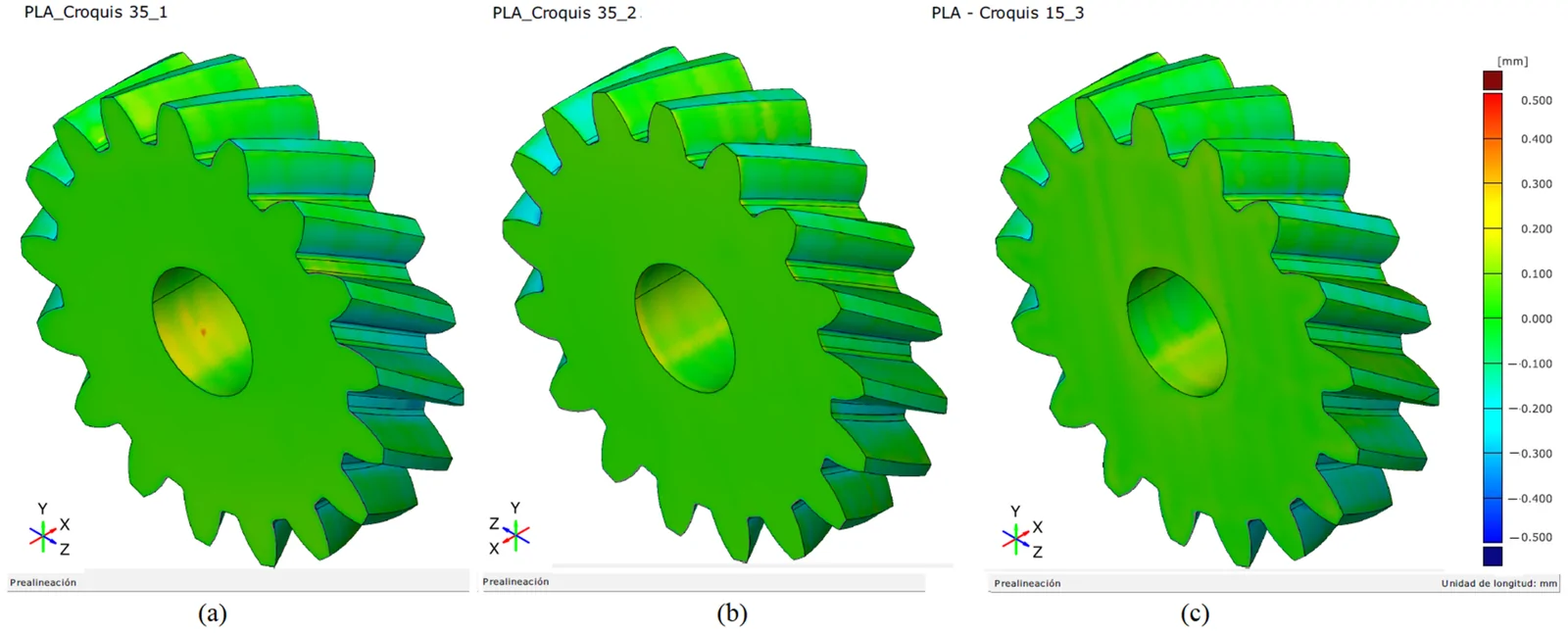
Scanned gear surfaces of PLA specimens with *β* = 35°: (**a**) replicate 1, (**b**) replicate 2, and (**c**) replicate 3, obtained using ZEISS INSPECT software.

**Figure 20:**
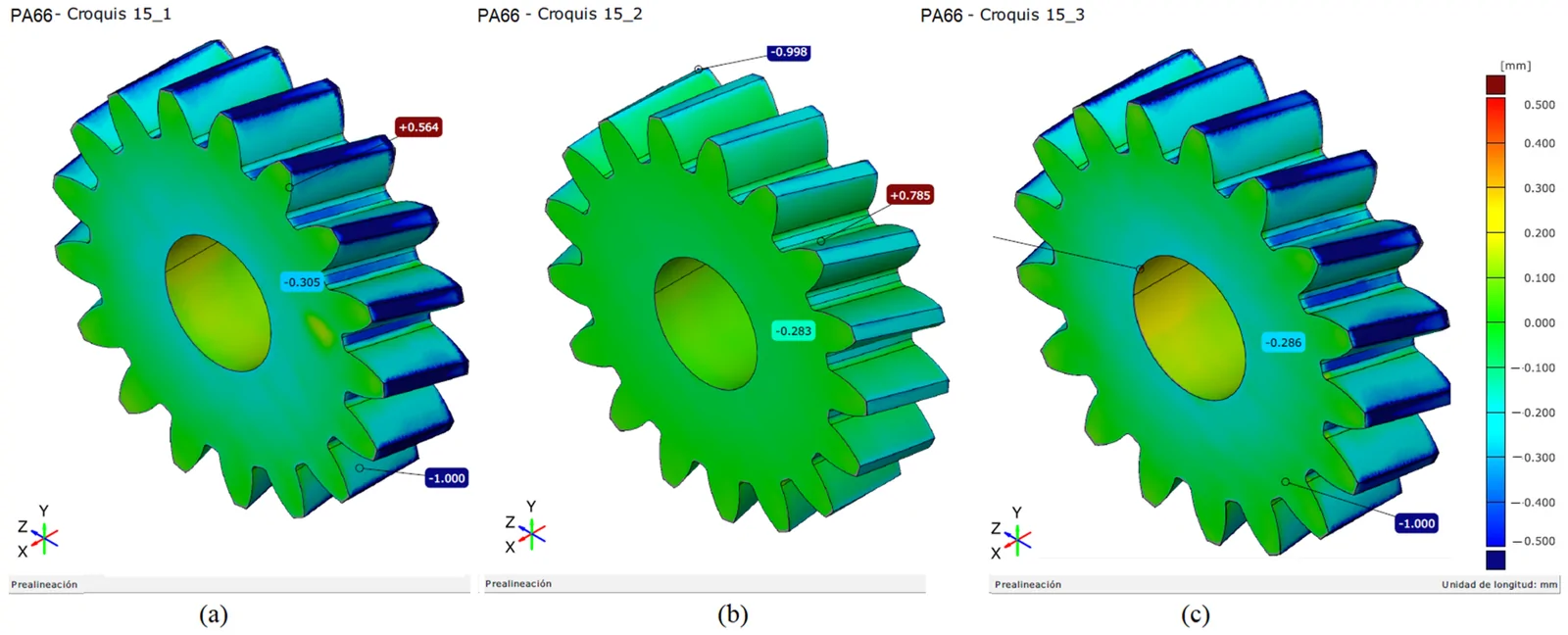
Scanned gear surfaces of PA66 specimens with *β* = 15°: (**a**) replicate 1, (**b**) replicate 2, and (**c**) replicate 3, obtained using ZEISS INSPECT software.

**Figure 21:**
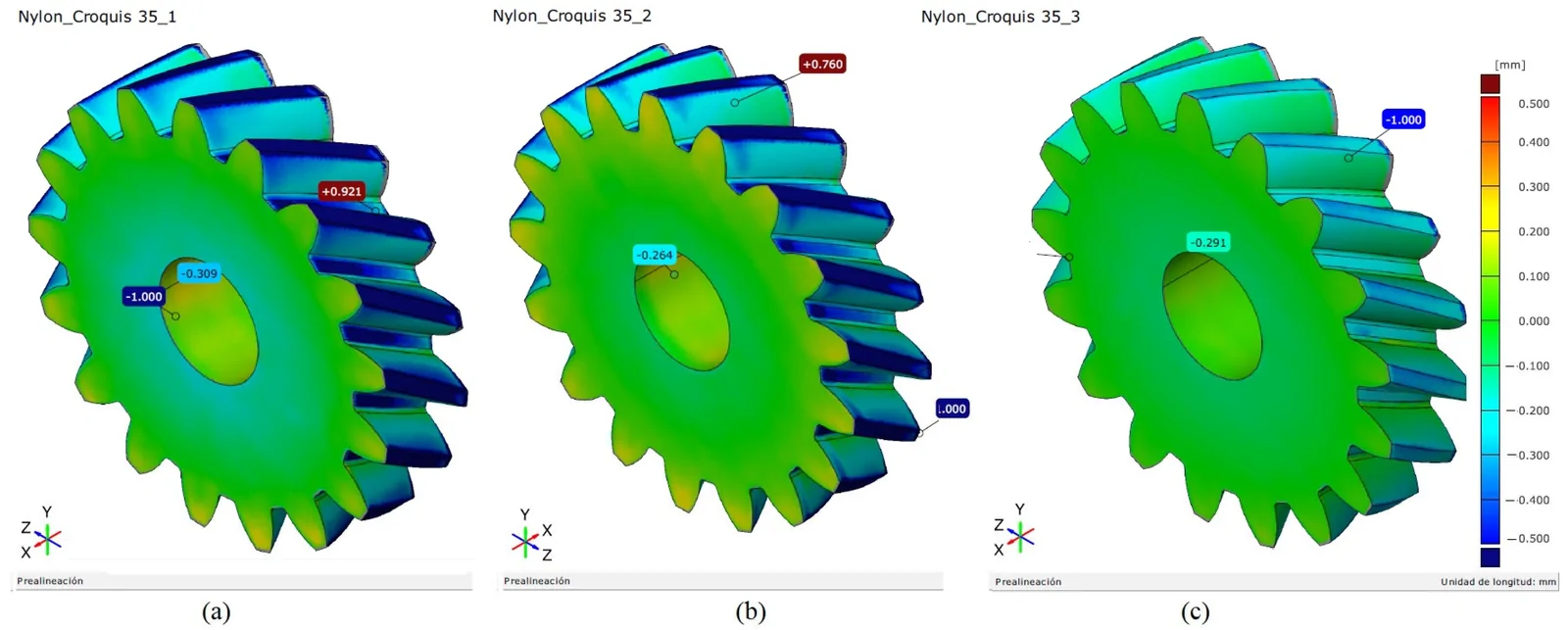
Scanned gear surfaces of PA66 specimens with *β* = 35°: (**a**) replicate 1, (**b**) replicate 2, and (**c**) replicate 3, obtained using ZEISS INSPECT software.

**Figure 22:**
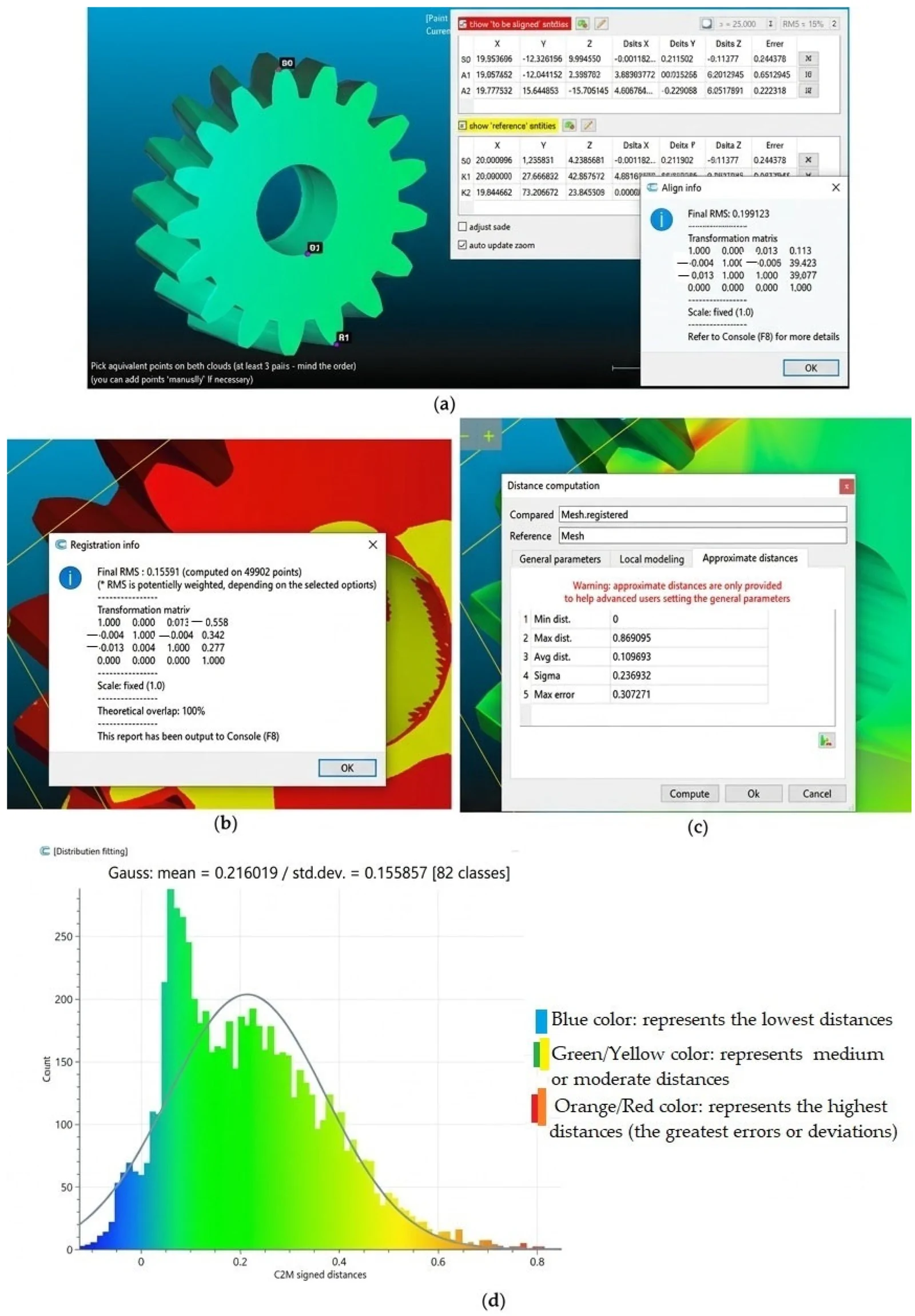
Dimensional comparison of the PLA gear specimen with a helix angle of *β* = 15° (replicate 1) using CloudCompare. Screenshots showing (**a**) selection of corresponding points for the initial alignment of the two gear meshes; (**b**) fine registration using the Iterative Closest Point (ICP) algorithm; (**c**) distance computation; and (**d**) statistical parameters.

**Figure 23:**
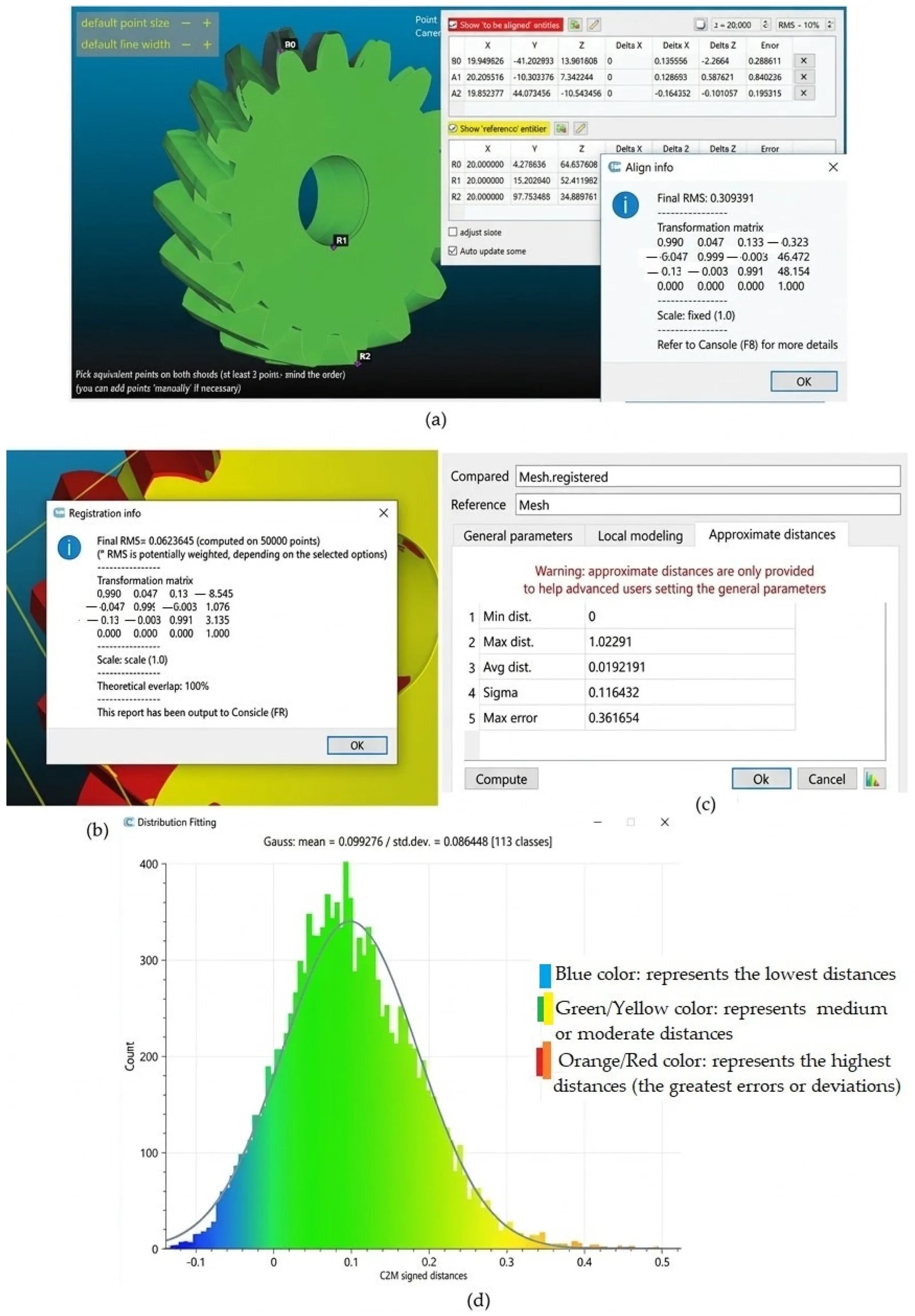
Dimensional comparison of the PLA gear specimen with a helix angle of *β* = 35° (replicate 1) using CloudCompare. Screenshots showing (**a**) selection of corresponding points for the initial alignment of the two gear meshes; (**b**) fine registration using the Iterative Closest Point (ICP) algorithm; (**c**) distance computation; and (**d**) statistical parameters.

**Figure 24:**
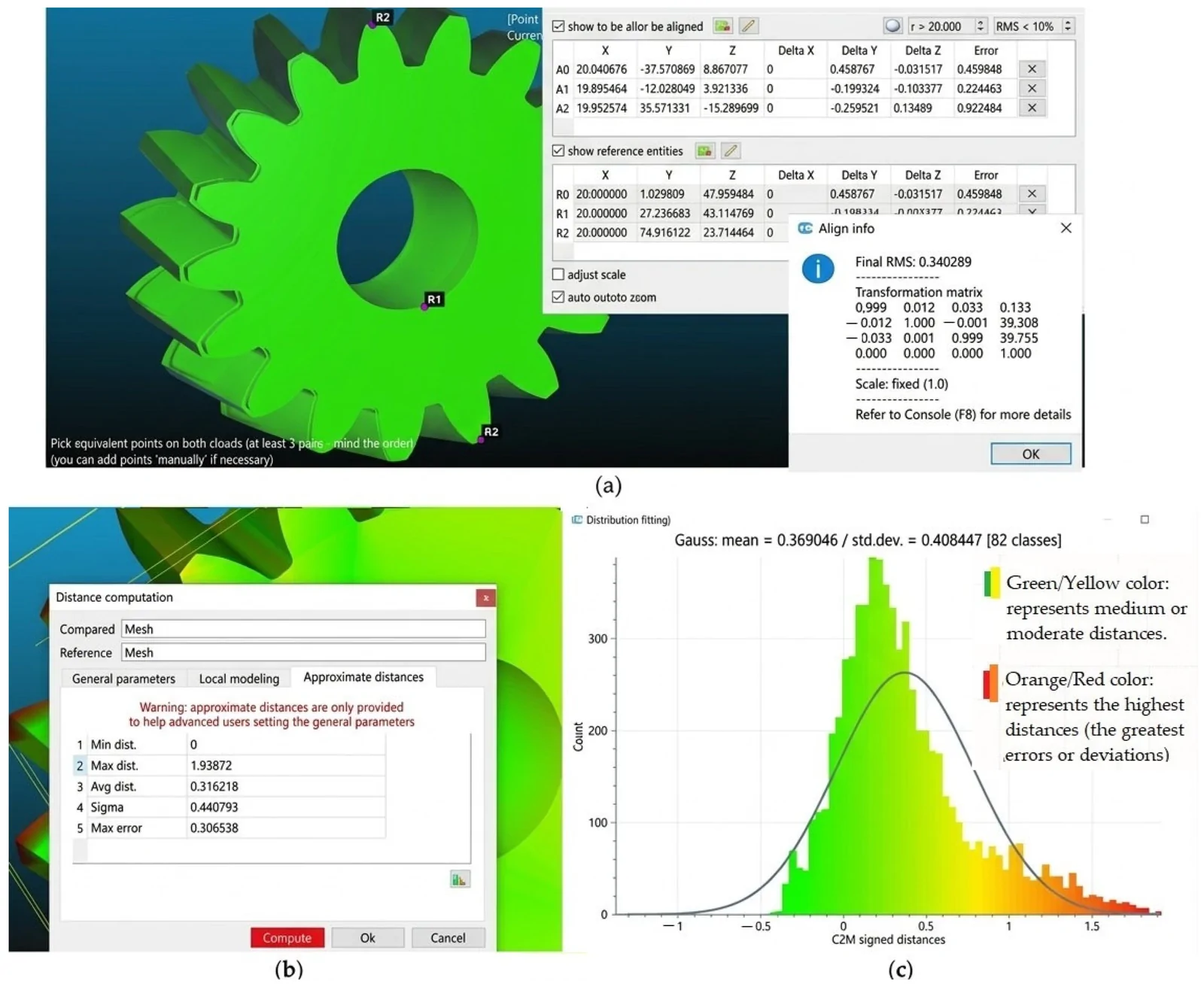
Dimensional comparison of the PA66 gear specimen with a helix angle of *β* = 15° (replicate 1) using CloudCompare. Screenshots showing (**a**) selection of corresponding points for the initial alignment of the two gear meshes, (**b**) distance computation, and (**c**) statistical parameters.

**Figure 25:**
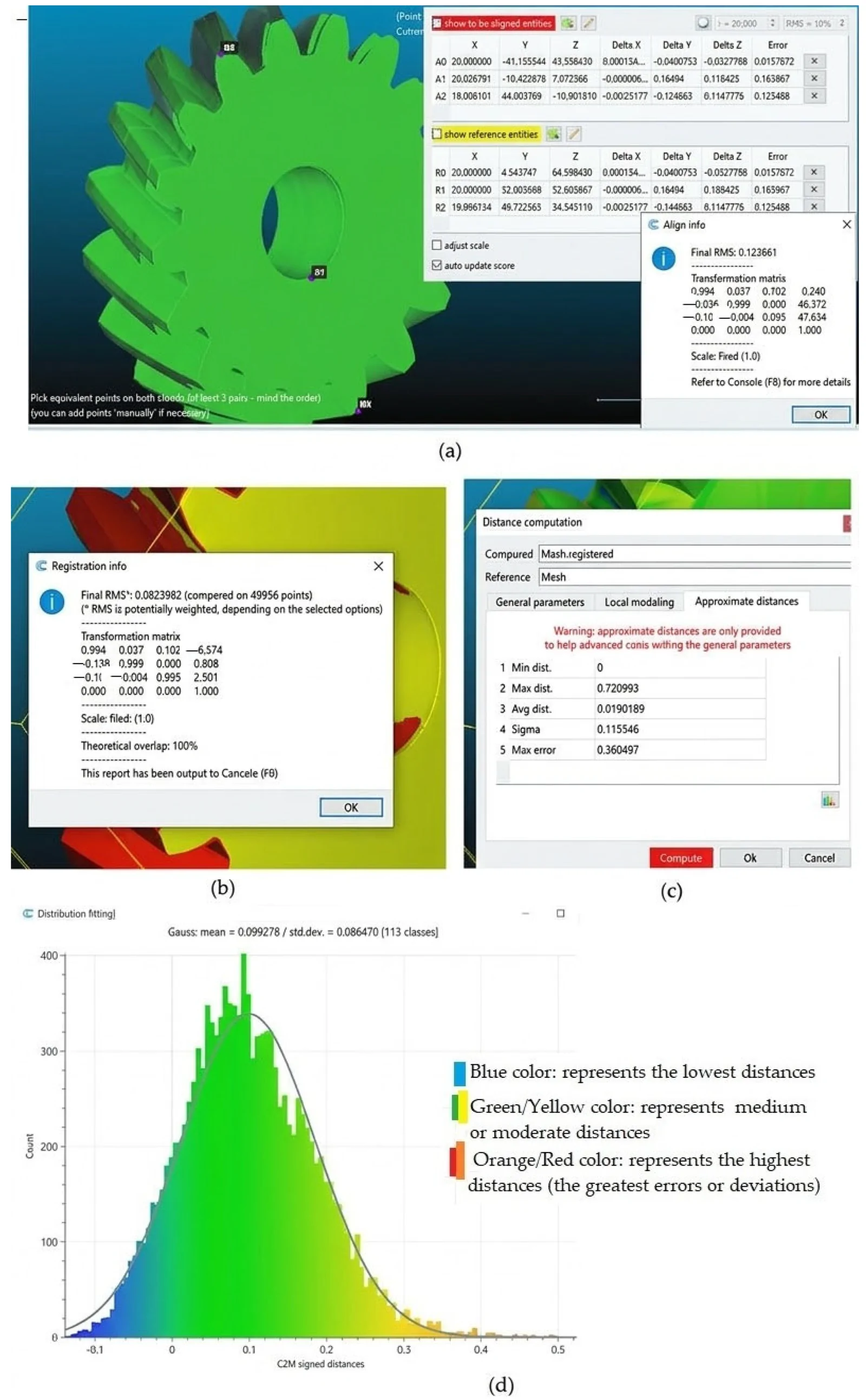
Dimensional comparison of the PA66 gear specimen with a helix angle of *β* = 35° (replicate 1) using CloudCompare. Screenshots showing (**a**) selection of corresponding points for the initial alignment of the two gear meshes, (**b**) fine registration using the Iterative Closest Point (ICP) algorithm, (**c**) distance computation, and (**d**) statistical parameters.

**Table 1:** Geometrical parameters of the three helical gear models designed.

Geometrical Parameters	Formula	Model 1	Model 2	Model 3
Module *m*_n_ [mm]	-	4	4	4
Number of teeth *z*	-	17	17	17
Helix angle *β* [°]	-	15	25	35
Normal pressure angle *α_n_* [°]	-	20	20	20
Transversal pressure angle α_t_ [°]	$\tan^{-1}\left(\tan\alpha_{n}/\cos\beta \right)$	20.65	21.88	23.96
Normal pitch *p*_n_ [mm]	*m*_n_ *· π*	12.57	12.57	12.57
Transversal pitch *p*_t_ [mm]	(*m*_n_ *· π*)*/*cosβ	13.01	13.87	15.35
Normal tooth thickness *s*_1n_ [mm]	(*m*_n_ · *π*)/2	6.28	6.28	6.28
Normal space width *s*_2n_ [mm]	(*m*_n_ *· π*)/2	6.28	6.28	6.28
Fillet radius *r*_b_ [mm]	*0.38·m_n_*	1.52	1.52	1.52
Addendum *h*_a_ [mm]	*m*_n_	4	4	4
Dedendum *h*_f_ [mm]	1.167 · *m*_n_	4.67	4.67	4.67
Tooth depth *h* [mm]	*h*_a_ + *h*_f_	8.67	8.67	8.67
Reference diameter *d* [mm]	(*m*_n_ · *z*)/cos *β*	70.40	75.03	83.01
Base diameter *d*_b_ [mm]	((*m*_n_ · *z*)/cos *β*) · cos α_t_	65.88	69.63	75.86
Root diameter *d*_f_ [mm]	*d* − 2 · *h*_f_	61.06	65.69	73.69
Tip diameter *d_a_* [mm]	*d* + 2 · *h*_a_	78.40	83.30	91.01

**Table 2:** Properties of PLA and PA66 filaments.

Properties	PLA/PHA	PA6/66
Density [g/cm^3^]	1.210–1.430	1.115
Glass transition temperature (Tg) [°C]	55	49
Melting temperature (Tm) [°C]	>155	195–197
Moisture absorption [%]	Low–moderate (Values not specified)	High (at 23 °C/50% relative moisture: ≈2.5–3.1%wt.)
Melt flow Index (MFI) [mm/s]	(190 °C/1.16 kg), 3.0–5.0 g/10 min.	Melt volume rate (MVR): 49.5 cm^3^/10 min

**Table 3:** Printing parameters used for PLA and PA66 materials.

Printing Parameters [Units]	PLA	PA66
Layer height [mm]	0.2	0.2
Print bed temperature [°C]	60	90
Printing temperature [°C]	195	255
Print speed [mm/s]	40	50
Infill rate [%]	50	20
Upper/Lower pattern	Zigzag	Zigzag
Infill pattern	Triangular	Triangular
Build orientation	Gear axis ∥ Z-axis	Gear axis ∥ Z-axis
Support structures	None	None
Cooling fan	Yes	No

**Table 4:** Geometrical parameters of the three models of helical gears designed.

Parameter [Units]	Numerical Value
Chordal height $\bar{h_{a}}$ [mm]	4.15
Chordal tooth thickness $\bar{s_{1}}$ [mm]	6.27

**Table 5:** Initial values for sector span *W* measurements.

Model	Sector Span of Teeth $z_{m}$	Sector Span *W* [mm]	Minimum Face Width $b_{min}\;[mm]$
Model 1 (*β* = 15°)	3	30.57	7.91
Model 2 (*β* = 25°)	3	30.78	13.01
Model 3 (*β* = 35°)	4	43.00	24.67

**Table 6:** Ideal diameter of rod values for each helical gear model printed.

Model	Ideal Diameter of Rod ${d^{\prime }}_{p}\;[mm]$
Model 1 (*β* = 15°)	6.91
Model 2 (*β* = 25°)	6.87
Model 3 (*β* = 35°)	6.82

**Table 7:** Theoretical diametral distance values for each 3D-printed helical gear model.

Model	Theoretical Diameter of Rod $d_{m}\;[mm]$
Model 1 (*β* = 15°)	86.17
Model 2 (*β* = 25°)	90.96
Model 3 (*β* = 35°)	99.23

**Table 8:** Tooth thickness $s_{1real}$ measurements.

Model, Material, Replicate		Measured Value (mm)	Mean	STD *	CI (95%)	Theoretical Value (mm)	Relative Error (%)
Model 1 *β* = 15° PA66	1	6.18		0.10			
	2	6.37	6.30		[6.04, 6.56]	6.28	0.32
	3	6.35					
Model 2 *β* = 25° PA66	1	6.41		0.07			
	2	6.34	6.34		[6.17, 6.51]	6.28	0.96
	3	6.27					
Model 3 *β* = 35° PA66	1	6.31		0.01			
	2	6.33	6.32		[6.29, 6.35]	6.28	0.58
	3	6.31					
Model 1 *β* = 15° PLA	1	6.30		0.01			
	2	6.29	6.30		[6.28, 6.31]	6.28	0.27
	3	6.30					
Model 2 *β* = 25° PLA	1	6.26		0.04			
	2	6.22	6.26		[6.16, 6.36]	6.28	0.32
	3	6.30					
Model 3 *β* = 35° PLA	1	6.15		0.06			
	2	6.27	6.21		[6.06, 6.36]	6.28	1.17
	3	6.20					

* Standard deviation.

**Table 9:** Sector span $W_{real}$ dimensions and key statistical parameters.

Model, Material, Replicate		Measured Value (mm)	Mean	STD *	CI (95%)	Theoretical Value (mm)	Relative Error (%)
Model 1 *β* = 15° PA66	1	30.73		0.02			
	2	30.69	30.71		[30.65, 30.76]	30.57	0.46
	3	30.70					
Model 2 *β* = 25° PA66	1	30.97		0.06			
	2	30.85	30.91		[30.76, 31.06]	30.78	0.42
	3	30.90					
Model 1 *β* = 15° PLA	1	31.20		0.02			
	2	31.20	31.19		[31.15, 31.23]	30.57	1.99
	3	31.17					
Model 2 *β* = 25° PLA	1	31.17		0.02			
	2	31.18	31.18		[31.15, 31.22]	30.78	1.28
	3	31.20					

* Standard deviation.

**Table 10:** Diametral distance $d_{mreal}$ dimensions and key statistical parameters.

Model, Material, Replicate		Measured Value (mm)	Mean	Stand. Dev. *	CI (95%)	Theoretical Value (mm)	Relative Error (%)
Model 1 *β* = 15° PA66	1	86.27				86.18	
	2	86.43	86.32	0.09	[86.09, 86.55]		0.16
	3	86.27					
Model 2 *β* = 25° PA66	1	90.64				90.96	
	2	90.35	90.59	0.22	[90.04, 91.15]		0.41
	3	90.79					
Model 3 *β* = 35° PA66	1	99.51				99.23	
	2	99.35	99.38	0.12	[99.09, 99.67]		0.15
	3	99.28					
Model 1 *β* = 15° PLA	1	86.32				86.18	
	2	86.18	86.26	0.07	[86.08, 86.44]		0.09
	3	86.28					
Model 2 *β* = 25° PLA	1	90.60				90.96	
	2	90.72	90.70	0.09	[90.48, 90.91]		0.29
	3	90.77					
Model 3 *β* = 35° PLA	1	99.42				99.23	
	2	99.24	99.24	0.18	[98.79, 99.69]		0.01
	3	99.06					

* Standard deviation.

## Data Availability

Additional data supporting reported results will be provided on request.

## Abbreviations

The following abbreviations are used in this manuscript:

AM	Additive Manufacturing
BJ	Binder Jetting
CAD	Computer-Aided Design
FDM	Fused Deposition Modeling
FFF	Fused Filament Fabrication
HPGL	Hewlett-Packard Graphics Language
MJ	Material Jetting
POM	Polyoxymethylene
PLA	Polylactic acid
SLA	Stereolithography
SLC	Sliced Layer Contour
SLS	Selective Laser Sintering
STL	Standard Triangle Language
